# New Genomes from the Congo Basin Expand History of CRF01_AE Origin and Dissemination

**DOI:** 10.1089/aid.2020.0031

**Published:** 2020-07-02

**Authors:** Dennis Maletich Junqueira, Eduan Wilkinson, Ana Vallari, Xianding Deng, Asmeeta Achari, Guixia Yu, Carole McArthur, Lazare Kaptue, Dora Mbanya, Charles Chiu, Gavin A. Cloherty, Tulio de Oliveira, Mary A. Rodgers

**Affiliations:** ^1^ Centro Universitário Ritter dos Reis-UniRitter, Porto Alegre, Brazil.; ^2^ KwaZulu-Natal Research Innovation Sequencing Platform (KRISP), University of KwaZulu-Natal, Durban, Republic of South Africa.; ^3^ School of Laboratory Medicine and Medical Science, College of Health Sciences, University of KwaZulu-Natal, Durban, Republic of South Africa.; ^4^ Abbott Diagnostics, Infectious Disease Research, Abbott Park, Illinois, USA.; ^5^ Department of Laboratory Medicine, University of California, San Francisco, California, USA.; ^6^ UCSF-Abbott Viral Diagnostics and Discovery Center, San Francisco, California, USA.; ^7^ School of Dentistry and School of Medicine, University of Missouri-Kansas City, Kansas City, Missouri, USA.; ^8^ Université des Montagnes, Bangangté, Cameroon.; ^9^ Université de Yaoundé I, Yaoundé, Cameroon.; ^10^ University of Bamenda, Bamenda, Cameroon.; ^11^ Division of Infectious Diseases, Department of Medicine, University of California, San Francisco, California, USA.; ^12^ Research Department of Infection, University College London, London, United Kingdom.

**Keywords:** HIV, CRF, surveillance, Congo, phylogenetics, molecular clock analysis

## Abstract

Although the first HIV circulating recombinant form (CRF01_AE) is the predominant strain in many Asian countries, it is uncommonly found in the Congo Basin from where it first originated. To fill the gap in the evolutionary history of this important strain, we sequenced near complete genomes from HIV samples with subgenomic CRF01_AE regions collected in Cameroon and the Democratic Republic of the Congo from 2001 to 2006. HIV genomes were generated from *N* = 13 plasma specimens by next-generation sequencing of metagenomic libraries prepared with spiked primers targeting HIV, followed by Sanger gap-filling. Genome sequences were aligned to reference strains, including Asian and African CRF01_AE sequences, and evaluated by phylogenetic and recombinant analysis to identify four CRF01_AE strains from Cameroon. We also identified two CRF02, one CRF27, and six unique recombinant form genomes (01|A1|G, 01|02|F|U, F|G|01, A1|D|01, F|G|01, and A1|G|01). Phylogenetic analysis, including the four new African CRF01_AE genomes, placed these samples as a bridge between basal Central African Republic CRF01_AE strains and all Asian, European, and American CRF01_AE strains. Molecular dating confirmed previous estimates indicating that the most recent common CRF01_AE ancestor emerged in the early 1970s (1968–1970) and spread beyond Africa around 1980 to Asia. The new sequences and analysis presented in this study expand the molecular history of the CRF01_AE clade, and are illustrated in an interactive Next Strain phylogenetic tree, map, and timeline at (https://nextstrain.org/community/EduanWilkinson/hiv-1_crf01).

## Introduction

CRF01_AE was the first circulating recombinant form (CRF) to be identified in the HIV type 1 (HIV-1) epidemic.^1,2^ The genome structure of this virus appeared to be a mosaic strain containing a mixture of subtype A and portions of sequences that did not cluster with other known subtypes of HIV-1 at the time. The non-A regions (portions of *vif*, *vpr*, *env*, *nef*, and 3′ long terminal repeat) were classified as subtype E, although a complete subtype E genome has not been identified to date. The earliest isolates of CRF01_AE were collected in 1990 in the Central African Republic (CAR).^2–4^ Molecular characterization of the envelope region from strains circulating in Africa in the 1990s indicated that genotype E accounted for 31% of 29 randomly selected samples collected in CAR,^4^ whereas subtype E was absent from studies conducted in Uganda, Zaire (now Democratic Republic of the Congo [DRC]), Malawi, and Tanzania.^5–8^

In contrast, HIV isolates collected from Thailand in the 1990s were dominated by subtype E envelope sequences.^9^ Once this “subtype” was officially recognized as CRF01_AE in the late 1990s, many molecular epidemiological surveys confirmed that CRF01_AE has continued to predominate the epidemics in Southeast Asia over the past three decades (reaching up to almost 82% among HIV-1 infections in the region).^10^ The spread of CRF01_AE has been tracked from Thailand to Vietnam, China, and ultimately a global dissemination.^11–14^ However, CRF01_AE has not extensively spread within Central Africa, with CRF01_AE infection having a prevalence of <1% in nearby Cameroon,^15^ <4% in DRC,^16,17^ <3% in Chad,^18,19^ <1% in the Republic of the Congo,^20–22^ and 6% in CAR^23^ in recent studies. However, the extent to which CRF01_AE may still be circulating within recombinant strains cannot be evaluated with subgenomic sequences reported in most of these studies.

Both subgenomic sequences and complete genomes have been used to estimate when CRF01_AE first emerged in Africa and became a key Asian strain. Bayesian analysis with subgenomic sequences estimated the origins of CRF01_AE in Central Africa to the 1960s by several models.^14^ More recently, analyses with four complete genome sequences have estimated that CRF01_AE first emerged in CAR in the 1970s and was introduced in Asia in the early 1980s (Ref.^12,13^). The evolutionary path of CRF01_AE between CAR and Asia has not been well characterized due to the limited number of only four CRF01_AE complete genome sequences ever produced from Africa, which could be a reflection of its low prevalence.

To address this gap, near-complete genome sequences were generated from *N* = 13 specimens collected in Cameroon and DRC carrying HIV strains identified as CRF01_AE by next-generation sequencing (NGS) and subgenomic Sanger sequencing. Phylogenetic analysis with references from CAR and Asia indicates that four new Cameroonian CRF01_AE strains sequenced herein provide a new molecular link between the two previously well-characterized major clades.

## Materials and Methods

### Study subjects

Thirteen plasma specimens were collected between 2001 and 2006 from HIV-1-positive individuals participating in viral diversity studies in Cameroon and the DRC (Table 1). The specimens came from blood donors or participants seeking voluntary testing as previously described.^16,24^ The DRC study was approved by the University of Missouri-Kansas City Research Board. The Cameroon study was approved by the Cameroon National Ethical Review Board, the Faculty of Medicine and Biomedical Science Institutional Review Boards (IRB), and the Ministry of Health of Cameroon.

**Table 1.** Sample Origin and Recombination Analysis Summary of Thirteen HIV-1 Sequences Isolated in Africa

Sample ID	Year	Country	Viral load (log copies/mL)	REGA		jpHMM	Simplot	BLAST		RAxML	Final
				Classification	Bootstrap strength			Classification	Identity		
260-27	2005	CM	5.32	NA	NA	A1/G/01/U	01/G/A	NA	90%	CRF01_AE	URF_A1/G/01
890-05	2006	CM	5.05	NA	NA	A1/G	A1/G	CRF02_AG	93%	CRF02_AG	CRF02_AG
A1188	2002	CM	4.25	NA	NA	CRF01/A1/G	CRF01/A1/G	CRF01_AE	92%	CRF01_AE	URF_01/A1/G
A1152	2002	CM	4.22	CRF02_AG	100	A1/G	A1/G	CRF02_AG	93%	CRF02_AG	CRF02_AG
A1549	2002	CM	4.11	NA	NA	A1/G/F2/01	CRF01/A1/F/G/U	URF_0222	89%	CRF01_AE	URF_01/02/F/U
CG-0422-02V	2002	DRC	NA	NA	NA	A1/D/01	A1/D/01	NA	90%	D	URF_A1/D/01
CG-0427a-02V	2002	DRC	NA	NA	NA	F1/G/01/U	F/G/01	F1	90%	F1	URF_F/G/01
CG-0070-01	2001	DRC	NA	NA	NA	F1/G/01/U	F/G/01	F1	89%	F1	URF_F/G/01
CG-0077-01	2003	DRC	NA	CRF27_cpx	100	G/H/C/J/01/A1/B	G/H/J/C/01/A1	CRF27_cpx	90%	CRF27_cpx	CRF27_cpx
U7957	2003	CM	NA	CRF01_AE	100	CRF01_AE	CRF01_AE^a^	CRF01_AE	92%	CRF01_AE	CRF01_AE
U8216	2003	CM	NA	CRF01_AE	100	CRF01_AE	CRF01_AE^a^	CRF01_AE	93%	CRF01_AE	CRF01_AE
234-40	2005	CM	4.14	CRF01_AE	100	CRF01_AE	CRF01_AE^a^	CRF01_AE	92%	CRF01_AE	CRF01_AE
1002-28	2006	CM	4.81	CRF01_AE	100	CRF01_AE	CRF01_AE^a^	CRF01_AE	92%	CRF01_AE	CRF01_AE

^a^ Reference sequences for the Simplot analyses are described in Supplementary Table S2.

01, CRF01_AE; BLAST, Basic Local Alignment Search Tool; CM, Cameroon; CRF, circulating recombinant form; DRC, Democratic Republic of the Congo; jpHMM, jumping profile Hidden Markov Model; NA, not assigned; RAxML, Randomized Axelerated Maximum Likelihood; REGA, software from Rega Institute in Central Belgium; URF, unique recombinant form.

### Amplification and sequencing

All near full-length nucleotide sequences of HIV-1 (HXB2: 94-9720) were amplified and sequenced from plasma viral RNA by a metagenomic sequencing with spiked primer enrichment (MSSPE) NGS approach.^25^ Gaps were filled in by supplemental Sanger sequencing.^16^ Briefly, nucleic acid was extracted from plasma according to the manufacturers' instructions using (i) the EZ1 Advanced XL system and EZ1 Virus Mini Kit (Qiagen, Hilden, Germany) for NGS or (ii) the RNA extraction protocol on an m2000sp system (Abbott Molecular, Des Plaines, IL) for Sanger sequencing. For NGS, random hexamer (RH) primers (Life Technologies) were spiked with HIV-specific primers.^25^ These primers were designed from 3,571 reference genomes, which cover genotypes M, N, O, and P of HIV-1 (Supplementary Table S1). A total of 798 primers of 13 nucleotides (464 forward and 334 reverse) were ordered and synthesized by Integrated DNA Technologies, Inc. (IDT, Coralville, IA). The HIV RNA extract was mixed with spiked primer (4 μM) plus RH (0.4 μM) in a 10:1 ratio and heated to 65°C for 5 min. The reverse transcription master mix (10 mL SuperScript III buffer, 5 mL dNTP of 12.5 mM, 2.5 mL DTT of 0.1 M, and 1 mL SuperScript III enzyme) was added to each sample and incubated at 25°C for 5 min, followed by 42°C for 30 min and 94°C for 2 min. Generated complementary DNA went through metagenomic library preparation using Illumina Nextera XT protocol to be analyzed on a HiSeq instrument (Illumina). Genome consensus sequences were generated using CLC Bio and Sanger sequences covering gaps were merged into the consensus using Sequencher v5.4.1 (Genecodes) as previously described.^16^ All open reading frames were annotated with SeqBuilder (DNAStar Laservene, v15) software. The Genbank accession numbers for the 13 HIV-1 genomes are MN116200-MN116212.

### Recombination analysis and sequence data

Recombination structures of all thirteen genomes were determined by five different detection methods: (i) REGA subtyping tool v3.0 (Ref.^26^); (ii) jumping profile Hidden Markov Model (jpHMM)-HIV^27^; (iii) Simplot v3.5.1 (Ref.^28^) under the Kimura 2-parameter model and a sliding window of 200 nucleotides increased by increments of 20 nucleotides; (iv) nucleotide Basic Local Alignment Search Tool (BLAST) to search for the most similar sequences; and (v) through phylogenetic reconstruction against a comprehensive dataset of 500 HIV-1 whole-genome sequences^16^ or through. The whole-genome phylogenetic reconstruction was performed in Randomized Axelerated Maximum Likelihood (RAxML)^29^ incorporating the best-fitting model of nucleotide substitution as determined in important quartets (IQ)-Tree^30^ and 1,000 bootstrap replicates^31^ that were used to infer transfer support for branches in the phylogeny.^32^ The final genome classification was defined based on the results of all five methods.

### Phylogenetic and phylogeographic reconstruction

Isolates identified as CRF01_AE in this study were analyzed against all whole-genome CRF01_AE sequences in the Los Alamos National Laboratory (LANL) HIV-1 sequence database (*n* = 382; accessed October 2018). First, we re-subtyped these reference sequences with REGA v3.0 (Ref.^26^) and jpHMM^27^ to insure the correct subtype assignment. Sequences presenting classification discrepancies from the Genbank information were excluded from the dataset (*n* = 11).

The final dataset was submitted to a Nextstrain build.^33^ Nextstrain consists of a suite of tools that take raw sequences (in fasta format) and associated metadata (e.g., time, country, publications, authors, and/or journals) as input. In short, Nextstrain performs a sequence alignment of the input data in multiple sequence alignment based on Fast Fourier transform (MAFFT),^34^ which in turn is used to infer a maximum likelihood. For the purpose of this study, the maximum likelihood (ML) tree topology was inferred in RAxML using the general time-reversible (GTR)+G+I model of nucleotide substitution.^35^ The resulting tree topology is then transformed into a dated phylogeny where the branches correspond to units of real calendar time using a least squared dating approach.^36^ In turn, the dated phylogeny is used to perform ancestral state reconstruction to infer the probable location of internal nodes in the phylogeny using a marginal likelihood approach. Finally, the time-scaled tree and the spatial reconstruction are then visualized together in a web browser with auspice.^33^

### Phylodynamic reconstruction

Demographic history of the CRF01_AE epidemic was examined under a Bayesian Markov Chain Monte Carlo approach as implemented in BEAST v.1.8.4 (Ref.^37^). First, a phylogenetic tree of the CRF01_AE whole-genome sequences (*n* = 4) was aligned with 371 whole-genome CRF01_AE reference sequences from LANL in MAFFT v7.0 (Ref.^34^). The subsequent alignment was used to construct an ML tree topology in RAxML v8.0 under the GTR+G+I model of nucleotide substitution and 1,000 bootstrap replicates. The resulting phylogeny was manually inspected in FigTree^38^ and highly monophyletic clades—defined as containing three or more sequences from the same country and with good branch support (>0.95)—were pruned with the ape package in R^39^ to only one sequence, preferably keeping the oldest isolate in the clade. The pruned phylogeny (*N* = 179) was tested for good clock-like signal in Tempest v1.5.1 using the sampling dates and root-to-tip divergence.^40^ An additional 61 sequences were removed as outliers resulting in a final R^2^ of 0.903 and a slope rate of ∼3.22 × 10^−3^ mutations per site per year, and with an *x*-intercept around 1969.7 (Supplementary Fig. S1).

This dataset was submitted to a Bayesian analysis incorporating the best-fitted nucleotide substitution model (GTR +4Γ+I+F) as determined in IQ-Tree^41^ with an uncorrelated lognormal relaxed molecular clock^42^ and various coalescent tree priors (e.g., skyride, skygrid, and skyline, constant and exponential growth). Bayes factor comparison between various coalescent tree priors consistently favored the use of the skyride coalescent tree prior.^43^ The molecular clock was calibrated using the sampling dates of the sequences under a uniform-distributed prior for the evolutionary rate according to previous simulations. After 5 × 10^8^ steps, convergence was checked using Tracer v1.6 discarding the first 50% of values for burn-in.^44^ Following the discarding of the burn-in, the remaining posterior parameters and trees were used to reconstruct the demographic growth of the global HIV-1 CRF01_AE linage through time.

## Results

### Sequence generation

A set of *N* = 13 specimens with subgenomic sequences classified as CRF01 was identified from previous viral diversity studies conducted in Cameroon and DRC for complete genome sequencing. A range of 4.11–5.32 log copies/ml HIV RNA was detected among samples with previous viral load results available (*N* = 7). NGS of all 13 samples using the MSSPE method yielded complete or near-complete genomes of at least 8,800 nucleotides in length. Full coverage was achieved for *N* = 8 samples and >90% coverage for *N* = 5 (A1549, 1002-28, 234-40, A1152, and A1188), which were filled in further by Sanger sequencing. Average read depths for each genome ranged from 14 to 18,899 × and the number of reads mapping to the consensus ranged from 1,060 to 1,744,672.

### Recombination analysis

**FIG. 1.**
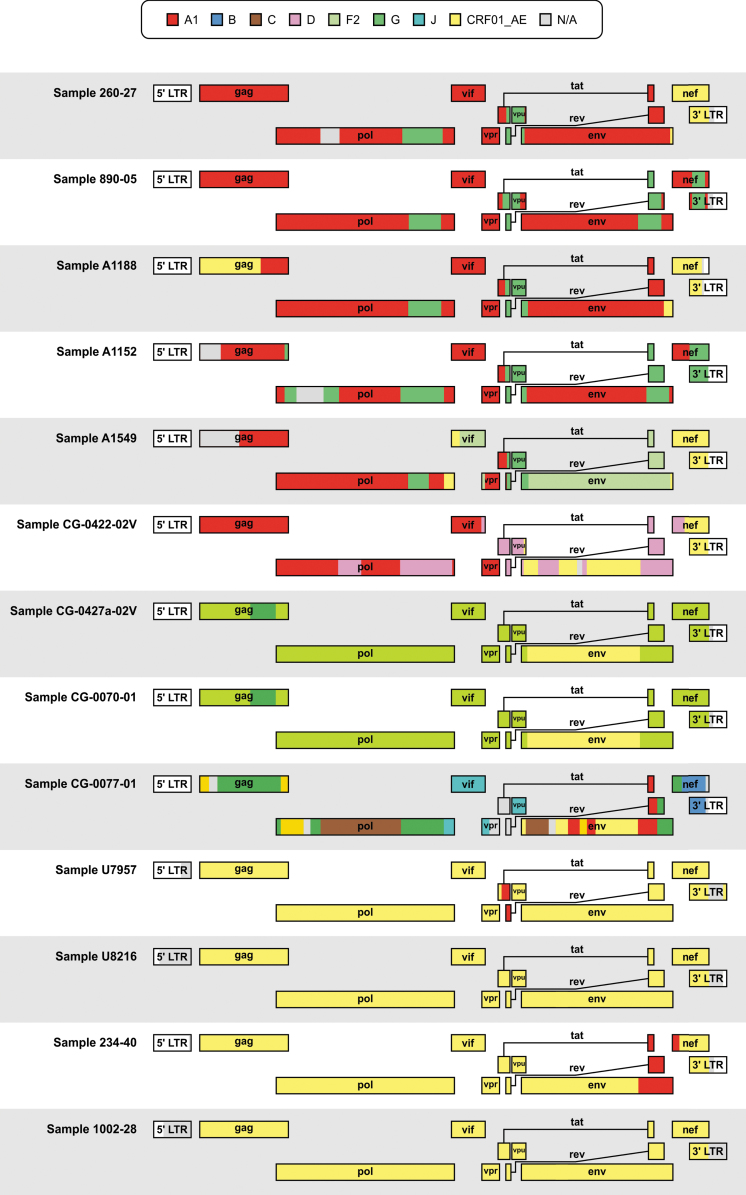
Schematic representation of jpHMM recombination results for thirteen HIV samples collected between 2001 and 2006 from HIV-1-positive individuals in Cameroon and the DRC samples isolated in the Congo Basin, Africa. Viral subtypes were denoted by *different colors* according to the legend. The *gray* N/A portions correspond to unclassifiable regions. CRF, circulating recombinant form; DRC, Democratic Republic of the Congo; jpHMM, jumping profile Hidden Markov Model; LTR, long terminal repeat; N/A, not assigned.

Genome-wide recombination analyses of whole-genome sequences were performed to determine the viral subtype and to detect evidence of intersubtype recombination. Despite minor differences, all five methods we employed to characterize the genome profiles of the whole-genome sequences broadly led to similar results (Table 1). Based on consensus agreement between the five different methods, we identified four CRF01_AE isolates (U7957, U8216, 234-40, and 1002-28) and six unique recombinant forms (260-27, A1188, A1549, CG-0422-02V, CG-0427a-02V, and CG-0070-01) that included portions of genomic regions assigned to CRF01_AE (Fig. 1). Of the remaining three whole-genome sequences, two were classified as CRF02_AG (890-05 and A1152) and one as belonging to CRF27_cpx (CG-0077-01).

### Phylogenetic and phylogeographic reconstruction

**FIG. 2.**
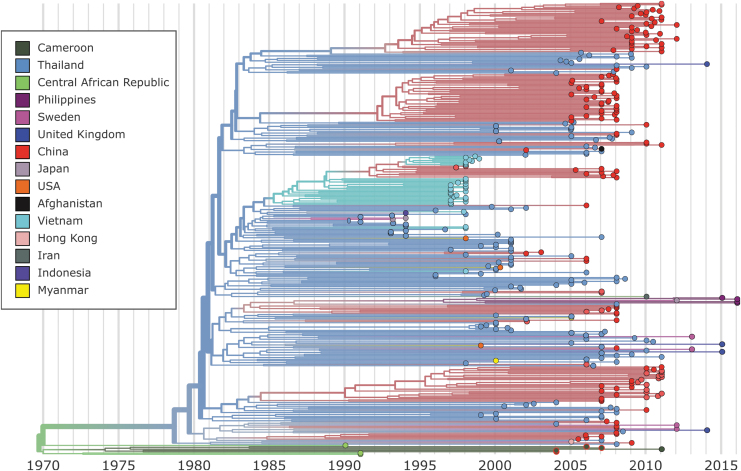
Nextstrain maximum-likelihood phylogenetic analysis of CRF01_AE whole-genome sequences. Tree branches are colored according to sampling origin. The sequences described in this study are located at the base of the tree identified by a *red contour*.

**FIG. 3.**
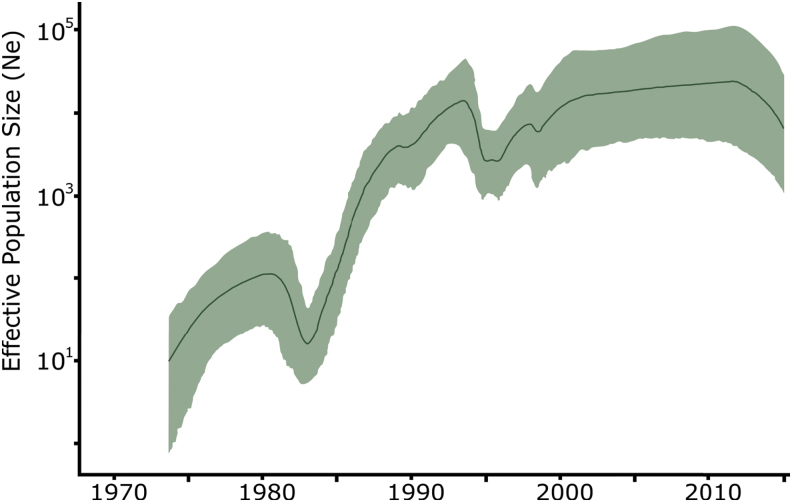
Temporal dynamics of CRF01_AE spread from Africa. Bayesian skygrid estimates of past population dynamics for CRF01_AE were plotted. The *left y*-axis represents the effective number of infections (Ne) and the *x*-axis the time.

**FIG. 4.**
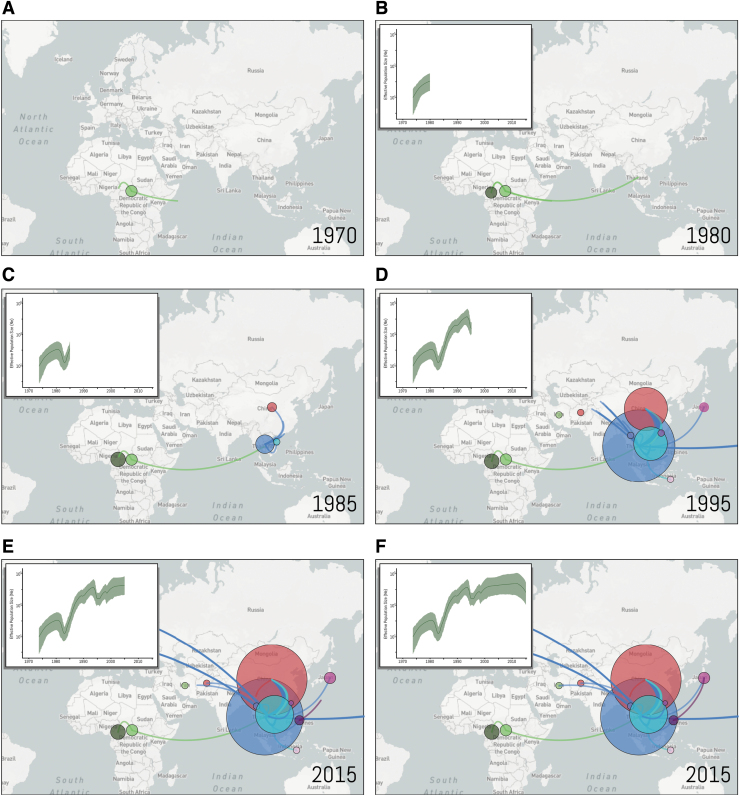
Nextstrain geographical view of CRF01_AE temporal dynamics. The geographical locations of isolates at each snapshot in time on the Bayesian skygrid are depicted with links between related strains indicated by a line. Lines between countries represent putative HIV transitions between regions and circle diameters are proportional to the square root of the number of branches that maintain the same location state at each time-point. Estimates of past population dynamics for CRF01_AE are plotted on the maps from **A** to **F**.

Only CRF01_AE sequences were submitted to further phylogenetic analysis. The four CRF01_AE strains isolated in this study were aligned to 371 CRF01_AE sequences from LANL, after a filtering step to achieve a more equitable temporal and geographic distribution as implemented in Nextstrain (final alignment; *n* = 358). Our phylogenetic and evolutionary analysis of near full-length CRF01_AE sequences in Nextstrain indicates that HIV-1 CRF01_AE arose in Africa in the early 1970s (1968–1970) (Figs. 2–4). As expected, our isolates grouped at the base of the tree with four other samples from Central Africa (three from the CAR and one from Cameroon) (Fig. 2). This pattern suggests that Cameroon has a secondary role in the African CRF01_AE epidemic and was seeded by Central African viruses around 1974 (1973–1975). The grouping of the four sequences in the base of the tree suggests that the current epidemic in Africa was directly derived from the primary CRF01_AE strains. The estimated evolutionary rate for this dataset was 1.5 × 10^−3^ nucleotide substitutions/site/year.

After almost 10 years of localized transmissions, the first divergence from the African strains was estimated to have occurred in the early 1980s (1980–1981) by the introduction of the virus in Thailand. It seems that only one or a few strains were imported from Africa and reached Asia. Thai sequences were found to be dispersed throughout the tree, structuring the basal positions to several clusters (Fig. 2). This pattern suggests that this lineage is likely to be the ancestor of all CRF01_AE strains in Asia (Fig. 4). Specifically, Thai sequences seeded the epidemics in China, Myanmar, the Philippines, Indonesia, the United States, The United Kingdom, and Sweden (Fig. 4). The epidemic in China that started in 1984 was driven by multiple independent lineages, most of them imported from Thailand. Thai sequences fall within the base of four major Chinese clusters and point to independent introductions of this strain into China. The Chinese epidemic was also seeded by strains from Vietnam in the mid-1990s (Fig. 2). In addition, Chinese sequences were also found dispersed throughout the tree lying next to Thai sequences indicating extensive interaction between the two epidemics. The evolutionary history of the CRF01_AE strains as they exited Africa and were introduced to Asia, Europe, and the Americas is illustrated in an interactive phylogenetic tree, map, and timeline at https://nextstrain.org/community/EduanWilkinson/hiv-1_crf01.

### Phylodynamics of CRF01_AE

We further investigated the past population dynamics of CRF01_AE using 179 sequences under a Bayesian skyride model (Fig. 3). Consistent with the ML results, we estimated the time of the most recent common ancestor of CRF01_AE to be around 1971 (95% highest posterior density (HPD): 1970–1973) (Supplementary Fig. S2). The estimate of the effective number of HIV-1 infections through time shows an exponential increase in the viral population between 1971 and 1980, period in which the virus was still circulating in Africa (Fig. 4). The introduction of CRF01_AE in Asian countries is followed by a short decrease in the CRF01_AE population probably due to the unsustained number of dead-end infections (Figs. 3 and 4). After 1985, the number of new CRF01_AE infections exponentially increases, reaching a plateau after the 2000s. More recently, we observed a decrease in these estimates; however, the 95% Bayesian confidence interval prevents us to confirming this hypothesis.

## Discussion

The four African CRF01_AE genomes presented in this study provide further resolution for the epidemiology of the CRF01_AE clade and provide a new genetic link between the African and Asian CRF01_AE clades of the HIV-1 pandemic. First, phylogenetic analysis with the new Cameroonian CRF01_AE strains confirmed the African origin of CRF01_AE.^12–14^ Likewise, molecular dating with these strains was consistent with previous estimates of emergence of CRF01_AE in Africa in the early 1970s and in Asia in the early 1980s (Refs.^12,13^). CRF01_AE appears to have originated in CAR where it spread to Cameroon and other countries in central Africa, although limited sampling from African countries prevents further resolution of the spread of CRF01_AE in the region. After ∼10 years of localized transmission in central Africa, CRF01_AE spread to Thailand and onward to other Asian countries (Fig. 4). These results suggest a scenario of multiple introductions into epidemiologically linked, high-risk groups, primarily heterosexual, people who inject drugs and man who have sex with man related clusters.

The four CRF01_AE genomes from Cameroon were identified among a larger set of *N* = 13 genomes that were previously classified as CRF01_AE based on sequencing of smaller subgenomic regions. However, the detection of recombination in the majority of these strains (*N* = 9) highlights the limitations of subgenomic sequences for classifying strains. Surprisingly, three recombinant genomes were identified among these that were classified as CRF02_AG or CRF27_cpx (Table 1). This finding is likely explained by similarities in the regions found in both CRF01_AE and CRF01_AG, as well as the presence of CRF01_AE in the complex recombinant pattern of CRF27_cpx.^4,45,46^ These unexpected classifications confirm that full-length sequences provide the most accurate classification. Furthermore, the identification of mostly recombinant genomes in this dataset indicates that reliance upon subgenomic sequences alone can lead to underestimation of the amount of HIV recombination in a population. This is especially relevant for estimating the true prevalence of CRF01_AE, which could be skewed by subgenomic sequences from unrecognized recombinant genomes. Although CRF01_AE is rare in the Congo Basin, it is likely that its prevalence is actually much lower than has been estimated due to subgenomic sequence limitations. Interestingly, CRF01_AE has recently been found at dramatically higher rates in Kenya in 2008 and 2015 (14%–25%),^47,48^ although with subgenomic sequence classifications. Whether these strains represent a resurgence of CRF01_AE or a CRF01_AE-containing recombinant in East Africa remains to be seen.

The phylodynamic reconstruction with a skyride tree prior and the addition of more African sequences led to a more refined demographic reconstruction of this HIV-1 clade. The reconstruction suggests a small decrease in the effective population size in the early 1980s with another dip in the mid 1990s. The first followed the transmission from Africa to Asia and may relate to this migration event, while the second dip in the effective population size may very well be due to behavioral changes in Thailand at the outbreak of the epidemic in that country.^49^

We anticipate that further sampling and sequencing of global CRF01_AE strains will provide additional resolution for the molecular and geographical epidemiology presented herein. To accommodate these future updates, the interactive CRF01_AE Next Strain phylogenetic tree, map, and timeline provide an ongoing resource for tracking this HIV clade. As more strains are sequenced, they can easily be merged with the CRF01_AE Next Strain dataset to enable comprehensive monitoring of this clade in real time.

## Sequence Data

The Genbank accession numbers for the 13 HIV-1 genomes are MN116200–MN116212.

## Supplementary Material

Supplemental data

Supplemental data

Supplemental data

Supplemental data
