# Supplementary material for: New Genomes from the Congo Basin Expand History of CRF01_AE Origin and Dissemination
Source: AIDS Res Hum Retroviruses. 2020 Jul 2;36(7):574–82. doi: 10.1089/aid.2020.0031 (PMC7398440; doi:10.1089/aid.2020.0031)
Supplement: Supplemental data [file Supp_Table2.pdf]

Supplementary Table S2. List of Reference Sequences Used for the Simplot Recombination Analysis of Putative CRF01_AE Sequences (U7957, U8216, 234-40, and 1002-28) Described in This Study

Subtype	*Country*	*Year*	*Access code*
A1	Australia	2003	DQ676872
A1	Rwanda	1992	AB253421
A1	Uganda	1992	AB253429
A2	The DRC	1997	AF286238
A2	Cameroon	2001	GU201516
A2	Cyprus	1994	AF286237
G	Belgium	1996	AF084936
G	Kenya	1993	AF061641
G	Nigeria	1992	U88826
G	Portugal	NA	AY612637
H	Belgium	1993	AF190127
H	Belgium	1993	AF190128
H	Central African Republic	1990	AF005496
H	United Kingdom	2000	FJ711703
J	The DRC	1997	EF614151
J	Cameroon	2004	GU237072
J	Sweden	1993	AF082394
K	The DRC	1997	AJ249235
K	Cameroon	1996	AJ249239
CRF01_AE	Afghanistan	2007	GQ477441
CRF01_AE	China	2005	GU564221
CRF01_AE	Thailand	1990	U54771
CRF02_AG	Cameroon	1999	AY271690
CRF02_AG	Liberia	NA	AB485636
CRF02_AG	Nigeria	NA	L39106

CRF, circulating recombinant form; DRC, Democratic Republic of the Congo; NA, not assigned.
