# Supplementary material for: New Genomes from the Congo Basin Expand History of CRF01_AE Origin and Dissemination
Source: AIDS Res Hum Retroviruses. 2020 Jul 2;36(7):574–82. doi: 10.1089/aid.2020.0031 (PMC7398440; doi:10.1089/aid.2020.0031)
Supplement: Supplemental data [file Supp_Table1.docx]

Supplementary Table S1. Seven Hundred Ninety-Eight 13 Nucleotide-Long Primers (464 Forward and 334 Reverse) for Amplification of HIV Groups M, N, O, and P

| Primer ID | Sequence |
| --- | --- |
| HIV_all_2016LANL_WestAfricanGroupM_3571seqs_140188_F_GC_0.69_avg_30.1945_std_6.63121_Tm_41.56 | GTGCGAGAGCGTC |
| HIV_all_2016LANL_WestAfricanGroupM_3571seqs_136344_F_GC_0.54_avg_30.1945_std_6.63121_Tm_35.25 | AACAGATGGCAGG |
| HIV_all_2016LANL_WestAfricanGroupM_3571seqs_132842_F_GC_0.46_avg_30.1945_std_6.63121_Tm_32.10 | ATCACTCTTTGGC |
| HIV_all_2016LANL_WestAfricanGroupM_3571seqs_129405_F_GC_0.46_avg_30.1945_std_6.63121_Tm_32.10 | TGATGACAGCATG |
| HIV_all_2016LANL_WestAfricanGroupM_3571seqs_126129_F_GC_0.31_avg_30.1945_std_6.63121_Tm_25.79 | TCAACATAATTGG |
| HIV_all_2016LANL_WestAfricanGroupM_3571seqs_123023_F_GC_0.31_avg_30.1945_std_6.63121_Tm_25.79 | CTGAAAATCCATA |
| HIV_all_2016LANL_WestAfricanGroupM_3571seqs_120104_F_GC_0.46_avg_30.1945_std_6.63121_Tm_32.10 | GTACAAATGGCAG |
| HIV_all_2016LANL_WestAfricanGroupM_3571seqs_114324_F_GC_0.31_avg_30.1945_std_6.63121_Tm_25.79 | TGGACATATCAAA |
| HIV_all_2016LANL_WestAfricanGroupM_3571seqs_111509_F_GC_0.38_avg_30.1945_std_6.63121_Tm_28.95 | GTCTCCATAGAAT |
| HIV_all_2016LANL_WestAfricanGroupM_3571seqs_108745_F_GC_0.38_avg_30.1945_std_6.63121_Tm_28.95 | TAGTAACAGACTC |
| HIV_all_2016LANL_WestAfricanGroupM_3571seqs_105996_F_GC_0.46_avg_30.1945_std_6.63121_Tm_32.10 | ATCAACAGCTCCT |
| HIV_all_2016LANL_WestAfricanGroupM_3571seqs_103304_F_GC_0.38_avg_30.1945_std_6.63121_Tm_28.95 | TATGTAGGATCTG |
| HIV_all_2016LANL_WestAfricanGroupM_3571seqs_100698_F_GC_0.23_avg_30.1945_std_6.63121_Tm_22.64 | ATGCAAATGTTAA |
| HIV_all_2016LANL_WestAfricanGroupM_3571seqs_98167_F_GC_0.54_avg_30.1945_std_6.63121_Tm_35.25 | TGGAGAGCAATGG |
| HIV_all_2016LANL_WestAfricanGroupM_3571seqs_95644_F_GC_0.54_avg_30.1945_std_6.63121_Tm_35.25 | CAGCAGAAACAGG |
| HIV_all_2016LANL_WestAfricanGroupM_3571seqs_93185_F_GC_0.31_avg_30.1945_std_6.63121_Tm_25.79 | TTTAACATGTGGA |
| HIV_all_2016LANL_WestAfricanGroupM_3571seqs_90750_F_GC_0.46_avg_30.1945_std_6.63121_Tm_32.10 | ATTGGGCAAGTCA |
| HIV_all_2016LANL_WestAfricanGroupM_3571seqs_88368_F_GC_0.31_avg_30.1945_std_6.63121_Tm_25.79 | AAATTATGGTACC |
| HIV_all_2016LANL_WestAfricanGroupM_3571seqs_86001_F_GC_0.54_avg_30.1945_std_6.63121_Tm_35.25 | TGGGGATACTTGG |
| HIV_all_2016LANL_WestAfricanGroupM_3571seqs_83662_F_GC_0.46_avg_30.1945_std_6.63121_Tm_32.10 | AATGAGACACCAG |
| HIV_all_2016LANL_WestAfricanGroupM_3571seqs_81350_F_GC_0.54_avg_30.1945_std_6.63121_Tm_35.25 | TATGGGGTACCTG |
| HIV_all_2016LANL_WestAfricanGroupM_3571seqs_79141_F_GC_0.69_avg_30.1945_std_6.63121_Tm_41.56 | CTCTCGACGCAGG |
| HIV_all_2016LANL_WestAfricanGroupM_3571seqs_77228_F_GC_0.31_avg_30.1945_std_6.63121_Tm_25.79 | AATTACCCTATAG |
| HIV_all_2016LANL_WestAfricanGroupM_3571seqs_75322_F_GC_0.69_avg_30.1945_std_6.63121_Tm_41.56 | GACCTGGAGGAGG |
| HIV_all_2016LANL_WestAfricanGroupM_3571seqs_73423_F_GC_0.46_avg_30.1945_std_6.63121_Tm_32.10 | CAGGATCAGAAGA |
| HIV_all_2016LANL_WestAfricanGroupM_3571seqs_71606_F_GC_0.38_avg_30.1945_std_6.63121_Tm_28.95 | TAAACATGTGGCA |
| HIV_all_2016LANL_WestAfricanGroupM_3571seqs_69801_F_GC_0.23_avg_30.1945_std_6.63121_Tm_22.64 | ATTCTAAAGTGTA |
| HIV_all_2016LANL_WestAfricanGroupM_3571seqs_68195_F_GC_0.31_avg_30.1945_std_6.63121_Tm_25.79 | AAGCACATTGTAA |
| HIV_all_2016LANL_WestAfricanGroupM_3571seqs_66591_F_GC_0.31_avg_30.1945_std_6.63121_Tm_25.79 | GAAAAGAATGAAC |
| HIV_all_2016LANL_WestAfricanGroupM_3571seqs_64998_F_GC_0.38_avg_30.1945_std_6.63121_Tm_28.95 | TCAAAGCAGTAAG |
| HIV_all_2016LANL_WestAfricanGroupM_3571seqs_63503_F_GC_0.54_avg_30.1945_std_6.63121_Tm_35.25 | CCACCTTTGCCTA |
| HIV_all_2016LANL_WestAfricanGroupM_3571seqs_62006_F_GC_0.38_avg_30.1945_std_6.63121_Tm_28.95 | TTAATTGTGGAGG |
| HIV_all_2016LANL_WestAfricanGroupM_3571seqs_60532_F_GC_0.46_avg_30.1945_std_6.63121_Tm_32.10 | ACTCACCTTTGTC |
| HIV_all_2016LANL_WestAfricanGroupM_3571seqs_59084_F_GC_0.54_avg_30.1945_std_6.63121_Tm_35.25 | GGAACTTCTGGGA |
| HIV_all_2016LANL_WestAfricanGroupM_3571seqs_57872_F_GC_0.62_avg_30.1945_std_6.63121_Tm_38.41 | CCACCTCTGCCTA |
| HIV_all_2016LANL_WestAfricanGroupM_3571seqs_56704_F_GC_0.23_avg_30.1945_std_6.63121_Tm_22.64 | AAACCATAATAGT |
| HIV_all_2016LANL_WestAfricanGroupM_3571seqs_54412_F_GC_0.23_avg_30.1945_std_6.63121_Tm_22.64 | TGCTATTGTAAAA |
| HIV_all_2016LANL_WestAfricanGroupM_3571seqs_53372_F_GC_0.46_avg_30.1945_std_6.63121_Tm_32.10 | CCAAAGCAGTAAG |
| HIV_all_2016LANL_WestAfricanGroupM_3571seqs_51436_F_GC_0.23_avg_30.1945_std_6.63121_Tm_22.64 | AATTATCCTATAG |
| HIV_all_2016LANL_WestAfricanGroupM_3571seqs_50531_F_GC_0.46_avg_30.1945_std_6.63121_Tm_32.10 | AGACAGGAACAGA |
| HIV_all_2016LANL_WestAfricanGroupM_3571seqs_49645_F_GC_0.15_avg_30.1945_std_6.63121_Tm_19.48 | GAATTTTTCTATT |
| HIV_all_2016LANL_WestAfricanGroupM_3571seqs_48781_F_GC_0.38_avg_30.1945_std_6.63121_Tm_28.95 | GAAACAGCATACT |
| HIV_all_2016LANL_WestAfricanGroupM_3571seqs_47953_F_GC_0.38_avg_30.1945_std_6.63121_Tm_28.95 | ACCATACCTAGTA |
| HIV_all_2016LANL_WestAfricanGroupM_3571seqs_47147_F_GC_0.31_avg_30.1945_std_6.63121_Tm_25.79 | AATAGACAGGTTA |
| HIV_all_2016LANL_WestAfricanGroupM_3571seqs_46360_F_GC_0.54_avg_30.1945_std_6.63121_Tm_35.25 | GCTGGTTTTGCGA |
| HIV_all_2016LANL_WestAfricanGroupM_3571seqs_45595_F_GC_0.46_avg_30.1945_std_6.63121_Tm_32.10 | TAAACTGGGCAAG |
| HIV_all_2016LANL_WestAfricanGroupM_3571seqs_44832_F_GC_0.38_avg_30.1945_std_6.63121_Tm_28.95 | TGTTGGAAATGTG |
| HIV_all_2016LANL_WestAfricanGroupM_3571seqs_44096_F_GC_0.38_avg_30.1945_std_6.63121_Tm_28.95 | TTTGTCAATACCC |
| HIV_all_2016LANL_WestAfricanGroupM_3571seqs_43361_F_GC_0.62_avg_30.1945_std_6.63121_Tm_38.41 | CGAGAGCGTCAGT |
| HIV_all_2016LANL_WestAfricanGroupM_3571seqs_42678_F_GC_0.31_avg_30.1945_std_6.63121_Tm_25.79 | TTTCGGGTTTATT |
| HIV_all_2016LANL_WestAfricanGroupM_3571seqs_42020_F_GC_0.54_avg_30.1945_std_6.63121_Tm_35.25 | GAAGCACTATGGG |
| HIV_all_2016LANL_WestAfricanGroupM_3571seqs_41364_F_GC_0.31_avg_30.1945_std_6.63121_Tm_25.79 | TAAATATGTGGCA |
| HIV_all_2016LANL_WestAfricanGroupM_3571seqs_40712_F_GC_0.31_avg_30.1945_std_6.63121_Tm_25.79 | TAGGAGATATAAG |
| HIV_all_2016LANL_WestAfricanGroupM_3571seqs_40060_F_GC_0.31_avg_30.1945_std_6.63121_Tm_25.79 | TAATAGTACAGCT |
| HIV_all_2016LANL_WestAfricanGroupM_3571seqs_39416_F_GC_0.23_avg_30.1945_std_6.63121_Tm_22.64 | TGTTACTGTAAAA |
| HIV_all_2016LANL_WestAfricanGroupM_3571seqs_38805_F_GC_0.46_avg_30.1945_std_6.63121_Tm_32.10 | GGAGGAGATATGA |
| HIV_all_2016LANL_WestAfricanGroupM_3571seqs_38206_F_GC_0.31_avg_30.1945_std_6.63121_Tm_25.79 | GAAAGATACCTAA |
| HIV_all_2016LANL_WestAfricanGroupM_3571seqs_37609_F_GC_0.54_avg_30.1945_std_6.63121_Tm_35.25 | TGTGGGTCACAGT |
| HIV_all_2016LANL_WestAfricanGroupM_3571seqs_37046_F_GC_0.54_avg_30.1945_std_6.63121_Tm_35.25 | CCAGCTGGTTATG |
| HIV_all_2016LANL_WestAfricanGroupM_3571seqs_36495_F_GC_0.46_avg_30.1945_std_6.63121_Tm_32.10 | GTCAGCCTAAAAC |
| HIV_all_2016LANL_WestAfricanGroupM_3571seqs_35956_F_GC_0.31_avg_30.1945_std_6.63121_Tm_25.79 | TAGTAACAGATTC |
| HIV_all_2016LANL_WestAfricanGroupM_3571seqs_35421_F_GC_0.23_avg_30.1945_std_6.63121_Tm_22.64 | ATATCTATGAAAC |
| HIV_all_2016LANL_WestAfricanGroupM_3571seqs_34895_F_GC_0.46_avg_30.1945_std_6.63121_Tm_32.10 | CACAGCAATTGGA |
| HIV_all_2016LANL_WestAfricanGroupM_3571seqs_34371_F_GC_0.38_avg_30.1945_std_6.63121_Tm_28.95 | TTAAGACAGCAGT |
| HIV_all_2016LANL_WestAfricanGroupM_3571seqs_33848_F_GC_0.15_avg_30.1945_std_6.63121_Tm_19.48 | AAAACAATAATAG |
| HIV_all_2016LANL_WestAfricanGroupM_3571seqs_33334_F_GC_0.38_avg_30.1945_std_6.63121_Tm_28.95 | TTGTAACACCTCA |
| HIV_all_2016LANL_WestAfricanGroupM_3571seqs_32829_F_GC_0.38_avg_30.1945_std_6.63121_Tm_28.95 | TAGAAATAGGGCA |
| HIV_all_2016LANL_WestAfricanGroupM_3571seqs_32340_F_GC_0.46_avg_30.1945_std_6.63121_Tm_32.10 | CAGGAGAAAGAGA |
| HIV_all_2016LANL_WestAfricanGroupM_3571seqs_31869_F_GC_0.38_avg_30.1945_std_6.63121_Tm_28.95 | TTGAAGAATCGCA |
| HIV_all_2016LANL_WestAfricanGroupM_3571seqs_31399_F_GC_0.23_avg_30.1945_std_6.63121_Tm_22.64 | AAAATAACATGGT |
| HIV_all_2016LANL_WestAfricanGroupM_3571seqs_30937_F_GC_0.46_avg_30.1945_std_6.63121_Tm_32.10 | TGTGGGTTACAGT |
| HIV_all_2016LANL_WestAfricanGroupM_3571seqs_30499_F_GC_0.31_avg_30.1945_std_6.63121_Tm_25.79 | TGCAGATGTTAAA |
| HIV_all_2016LANL_WestAfricanGroupM_3571seqs_30066_F_GC_0.46_avg_30.1945_std_6.63121_Tm_32.10 | TAGGCTTTCCAGT |
| HIV_all_2016LANL_WestAfricanGroupM_3571seqs_29637_F_GC_0.69_avg_30.1945_std_6.63121_Tm_41.56 | GAGCCAGCAGCAG |
| HIV_all_2016LANL_WestAfricanGroupM_3571seqs_28870_F_GC_0.31_avg_30.1945_std_6.63121_Tm_25.79 | AATCTGAAAACAG |
| HIV_all_2016LANL_WestAfricanGroupM_3571seqs_28511_F_GC_0.54_avg_30.1945_std_6.63121_Tm_35.25 | CAAAACCAGCAGG |
| HIV_all_2016LANL_WestAfricanGroupM_3571seqs_28152_F_GC_0.23_avg_30.1945_std_6.63121_Tm_22.64 | TATTTGCCATAAA |
| HIV_all_2016LANL_WestAfricanGroupM_3571seqs_27807_F_GC_0.54_avg_30.1945_std_6.63121_Tm_35.25 | CCAGAGCAGTAAG |
| HIV_all_2016LANL_WestAfricanGroupM_3571seqs_27469_F_GC_0.38_avg_30.1945_std_6.63121_Tm_28.95 | GGAGGAAATATGA |
| HIV_all_2016LANL_WestAfricanGroupM_3571seqs_27145_F_GC_0.15_avg_30.1945_std_6.63121_Tm_19.48 | TGTTATTGTAAAA |
| HIV_all_2016LANL_WestAfricanGroupM_3571seqs_26829_F_GC_0.23_avg_30.1945_std_6.63121_Tm_22.64 | TAAGAATGTATAG |
| HIV_all_2016LANL_WestAfricanGroupM_3571seqs_26514_F_GC_0.62_avg_30.1945_std_6.63121_Tm_38.41 | CAGAACCAGCAGG |
| HIV_all_2016LANL_WestAfricanGroupM_3571seqs_26201_F_GC_0.23_avg_30.1945_std_6.63121_Tm_22.64 | AATTTTTCTACTG |
| HIV_all_2016LANL_WestAfricanGroupM_3571seqs_25898_F_GC_0.15_avg_30.1945_std_6.63121_Tm_19.48 | AAAGAAAAATAGA |
| HIV_all_2016LANL_WestAfricanGroupM_3571seqs_25615_F_GC_0.31_avg_30.1945_std_6.63121_Tm_25.79 | TGCAAATGCTAAA |
| HIV_all_2016LANL_WestAfricanGroupM_3571seqs_25342_F_GC_0.38_avg_30.1945_std_6.63121_Tm_28.95 | TCACAAACAATGC |
| HIV_all_2016LANL_WestAfricanGroupM_3571seqs_25074_F_GC_0.31_avg_30.1945_std_6.63121_Tm_25.79 | TAATAGGAGACAT |
| HIV_all_2016LANL_WestAfricanGroupM_3571seqs_24813_F_GC_0.54_avg_30.1945_std_6.63121_Tm_35.25 | TAGGACCTACACC |
| HIV_all_2016LANL_WestAfricanGroupM_3571seqs_24552_F_GC_0.54_avg_30.1945_std_6.63121_Tm_35.25 | TCGTTTCAGACCC |
| HIV_all_2016LANL_WestAfricanGroupM_3571seqs_24292_F_GC_0.15_avg_30.1945_std_6.63121_Tm_19.48 | CTTAAATCATTAT |
| HIV_all_2016LANL_WestAfricanGroupM_3571seqs_24040_F_GC_0.54_avg_30.1945_std_6.63121_Tm_35.25 | CCAAAGCAGTGAG |
| HIV_all_2016LANL_WestAfricanGroupM_3571seqs_23790_F_GC_0.62_avg_30.1945_std_6.63121_Tm_38.41 | GGAGTAGGAGCAG |
| HIV_all_2016LANL_WestAfricanGroupM_3571seqs_23541_F_GC_0.54_avg_30.1945_std_6.63121_Tm_35.25 | CAGATGCATGAGG |
| HIV_all_2016LANL_WestAfricanGroupM_3571seqs_23294_F_GC_0.31_avg_30.1945_std_6.63121_Tm_25.79 | ATATAAGACAAGC |
| HIV_all_2016LANL_WestAfricanGroupM_3571seqs_23055_F_GC_0.46_avg_30.1945_std_6.63121_Tm_32.10 | TGGAGATACTTGG |
| HIV_all_2016LANL_WestAfricanGroupM_3571seqs_22824_F_GC_0.54_avg_30.1945_std_6.63121_Tm_35.25 | ATTGGGCAAGCCA |
| HIV_all_2016LANL_WestAfricanGroupM_3571seqs_22594_F_GC_0.31_avg_30.1945_std_6.63121_Tm_25.79 | TTGTAATACCTCA |
| HIV_all_2016LANL_WestAfricanGroupM_3571seqs_22371_F_GC_0.38_avg_30.1945_std_6.63121_Tm_28.95 | GTCAGCCAAAATT |
| HIV_all_2016LANL_WestAfricanGroupM_3571seqs_22150_F_GC_0.38_avg_30.1945_std_6.63121_Tm_28.95 | CACAGTAATTGGA |
| HIV_all_2016LANL_WestAfricanGroupM_3571seqs_21939_F_GC_0.23_avg_30.1945_std_6.63121_Tm_22.64 | AAAGGAAAATAGA |
| HIV_all_2016LANL_WestAfricanGroupM_3571seqs_21729_F_GC_0.38_avg_30.1945_std_6.63121_Tm_28.95 | AATGAAACACCAG |
| HIV_all_2016LANL_WestAfricanGroupM_3571seqs_21521_F_GC_0.23_avg_30.1945_std_6.63121_Tm_22.64 | AAATTATGGTATC |
| HIV_all_2016LANL_WestAfricanGroupM_3571seqs_21319_F_GC_0.46_avg_30.1945_std_6.63121_Tm_32.10 | GTAGGACAAGCAA |
| HIV_all_2016LANL_WestAfricanGroupM_3571seqs_21123_F_GC_0.23_avg_30.1945_std_6.63121_Tm_22.64 | TAGAAGAAATGAT |
| HIV_all_2016LANL_WestAfricanGroupM_3571seqs_20933_F_GC_0.38_avg_30.1945_std_6.63121_Tm_28.95 | CACTAACAGCATT |
| HIV_all_2016LANL_WestAfricanGroupM_3571seqs_20559_F_GC_0.15_avg_30.1945_std_6.63121_Tm_19.48 | AAAAACATAATAG |
| HIV_all_2016LANL_WestAfricanGroupM_3571seqs_20379_F_GC_0.31_avg_30.1945_std_6.63121_Tm_25.79 | GCGTCAATATTAA |
| HIV_all_2016LANL_WestAfricanGroupM_3571seqs_20200_F_GC_0.69_avg_30.1945_std_6.63121_Tm_41.56 | GAGGGAACCCGAC |
| HIV_all_2016LANL_WestAfricanGroupM_3571seqs_20023_F_GC_0.31_avg_30.1945_std_6.63121_Tm_25.79 | TGCTATTGTAAAC |
| HIV_all_2016LANL_WestAfricanGroupM_3571seqs_19846_F_GC_0.23_avg_30.1945_std_6.63121_Tm_22.64 | AATCTAAAAACAG |
| HIV_all_2016LANL_WestAfricanGroupM_3571seqs_19680_F_GC_0.31_avg_30.1945_std_6.63121_Tm_25.79 | TATGCATTAGGAA |
| HIV_all_2016LANL_WestAfricanGroupM_3571seqs_19514_F_GC_0.62_avg_30.1945_std_6.63121_Tm_38.41 | AGGACTCGGCTTG |
| HIV_all_2016LANL_WestAfricanGroupM_3571seqs_19348_F_GC_0.23_avg_30.1945_std_6.63121_Tm_22.64 | CTTAGATCATTAT |
| HIV_all_2016LANL_WestAfricanGroupM_3571seqs_19185_F_GC_0.54_avg_30.1945_std_6.63121_Tm_35.25 | GAGACCTTCAGAC |
| HIV_all_2016LANL_WestAfricanGroupM_3571seqs_19025_F_GC_0.38_avg_30.1945_std_6.63121_Tm_28.95 | TAGTGCAAAATGC |
| HIV_all_2016LANL_WestAfricanGroupM_3571seqs_18869_F_GC_0.54_avg_30.1945_std_6.63121_Tm_35.25 | GTCAGCCTACAAC |
| HIV_all_2016LANL_WestAfricanGroupM_3571seqs_18717_F_GC_0.69_avg_30.1945_std_6.63121_Tm_41.56 | TGGGACGCAGCAG |
| HIV_all_2016LANL_WestAfricanGroupM_3571seqs_18566_F_GC_0.15_avg_30.1945_std_6.63121_Tm_19.48 | TATTTGCTATAAA |
| HIV_all_2016LANL_WestAfricanGroupM_3571seqs_18416_F_GC_0.62_avg_30.1945_std_6.63121_Tm_38.41 | CAGCAGAGACAGG |
| HIV_all_2016LANL_WestAfricanGroupM_3571seqs_18266_F_GC_0.46_avg_30.1945_std_6.63121_Tm_32.10 | TCATTTCAGACCC |
| HIV_all_2016LANL_WestAfricanGroupM_3571seqs_18116_F_GC_0.54_avg_30.1945_std_6.63121_Tm_35.25 | CCAGCTGGTTTTG |
| HIV_all_2016LANL_WestAfricanGroupM_3571seqs_17967_F_GC_0.15_avg_30.1945_std_6.63121_Tm_19.48 | TAATAAATTGCAA |
| HIV_all_2016LANL_WestAfricanGroupM_3571seqs_17822_F_GC_0.46_avg_30.1945_std_6.63121_Tm_32.10 | TTTCCAGTCAGAC |
| HIV_all_2016LANL_WestAfricanGroupM_3571seqs_17681_F_GC_0.23_avg_30.1945_std_6.63121_Tm_22.64 | ATGATTTGTATGT |
| HIV_all_2016LANL_WestAfricanGroupM_3571seqs_17542_F_GC_0.46_avg_30.1945_std_6.63121_Tm_32.10 | GCACTGAAAGCAT |
| HIV_all_2016LANL_WestAfricanGroupM_3571seqs_17405_F_GC_0.46_avg_30.1945_std_6.63121_Tm_32.10 | TCAAAGCAGTGAG |
| HIV_all_2016LANL_WestAfricanGroupM_3571seqs_17268_F_GC_0.69_avg_30.1945_std_6.63121_Tm_41.56 | CCATCAGCCCCAC |
| HIV_all_2016LANL_WestAfricanGroupM_3571seqs_17132_F_GC_0.31_avg_30.1945_std_6.63121_Tm_25.79 | GAAAAGCATATTG |
| HIV_all_2016LANL_WestAfricanGroupM_3571seqs_17001_F_GC_0.46_avg_30.1945_std_6.63121_Tm_32.10 | TTGAAGACTCGCA |
| HIV_all_2016LANL_WestAfricanGroupM_3571seqs_16872_F_GC_0.31_avg_30.1945_std_6.63121_Tm_25.79 | AAGATACCATCAA |
| HIV_all_2016LANL_WestAfricanGroupM_3571seqs_16745_F_GC_0.54_avg_30.1945_std_6.63121_Tm_35.25 | TGGCATTTGGGTC |
| HIV_all_2016LANL_WestAfricanGroupM_3571seqs_16621_F_GC_0.46_avg_30.1945_std_6.63121_Tm_32.10 | TATGGGGACACTT |
| HIV_all_2016LANL_WestAfricanGroupM_3571seqs_16499_F_GC_0.38_avg_30.1945_std_6.63121_Tm_28.95 | ATGTAGGATCAGA |
| HIV_all_2016LANL_WestAfricanGroupM_3571seqs_16377_F_GC_0.62_avg_30.1945_std_6.63121_Tm_38.41 | CGAGGACTGTGGA |
| HIV_all_2016LANL_WestAfricanGroupM_3571seqs_16255_F_GC_0.38_avg_30.1945_std_6.63121_Tm_28.95 | GTAGGAAAAGCAA |
| HIV_all_2016LANL_WestAfricanGroupM_3571seqs_16137_F_GC_0.46_avg_30.1945_std_6.63121_Tm_32.10 | TATGGGGATACCT |
| HIV_all_2016LANL_WestAfricanGroupM_3571seqs_16020_F_GC_0.31_avg_30.1945_std_6.63121_Tm_25.79 | AAAAAGTGTTGCT |
| HIV_all_2016LANL_WestAfricanGroupM_3571seqs_15905_F_GC_0.23_avg_30.1945_std_6.63121_Tm_22.64 | TTTAATTGTAGAG |
| HIV_all_2016LANL_WestAfricanGroupM_3571seqs_15790_F_GC_0.15_avg_30.1945_std_6.63121_Tm_19.48 | TATATAATACAGT |
| HIV_all_2016LANL_WestAfricanGroupM_3571seqs_15676_F_GC_0.46_avg_30.1945_std_6.63121_Tm_32.10 | TGATTGTGTGGCA |
| HIV_all_2016LANL_WestAfricanGroupM_3571seqs_15563_F_GC_0.62_avg_30.1945_std_6.63121_Tm_38.41 | CCGGCTGGTTTTG |
| HIV_all_2016LANL_WestAfricanGroupM_3571seqs_15450_F_GC_0.38_avg_30.1945_std_6.63121_Tm_28.95 | TGGGAGTTTGTTA |
| HIV_all_2016LANL_WestAfricanGroupM_3571seqs_15337_F_GC_0.31_avg_30.1945_std_6.63121_Tm_25.79 | AAAATGACATGGT |
| HIV_all_2016LANL_WestAfricanGroupM_3571seqs_15226_F_GC_0.23_avg_30.1945_std_6.63121_Tm_22.64 | TAATAGTACATCT |
| HIV_all_2016LANL_WestAfricanGroupM_3571seqs_15116_F_GC_0.69_avg_30.1945_std_6.63121_Tm_41.56 | TGGGGCTGCTCTG |
| HIV_all_2016LANL_WestAfricanGroupM_3571seqs_15010_F_GC_0.23_avg_30.1945_std_6.63121_Tm_22.64 | TATAGGTTGATAA |
| HIV_all_2016LANL_WestAfricanGroupM_3571seqs_14904_F_GC_0.31_avg_30.1945_std_6.63121_Tm_25.79 | AGTATACTGCATT |
| HIV_all_2016LANL_WestAfricanGroupM_3571seqs_14799_F_GC_0.15_avg_30.1945_std_6.63121_Tm_19.48 | TAATTGATAGAAT |
| HIV_all_2016LANL_WestAfricanGroupM_3571seqs_14694_F_GC_0.46_avg_30.1945_std_6.63121_Tm_32.10 | CCACCTTTACCTA |
| HIV_all_2016LANL_WestAfricanGroupM_3571seqs_14590_F_GC_0.31_avg_30.1945_std_6.63121_Tm_25.79 | GTCAGTCAAAATT |
| HIV_all_2016LANL_WestAfricanGroupM_3571seqs_14488_F_GC_0.46_avg_30.1945_std_6.63121_Tm_32.10 | TCAGAGCAGTAAG |
| HIV_all_2016LANL_WestAfricanGroupM_3571seqs_14388_F_GC_0.15_avg_30.1945_std_6.63121_Tm_19.48 | AAATCATAATAGT |
| HIV_all_2016LANL_WestAfricanGroupM_3571seqs_14289_F_GC_0.31_avg_30.1945_std_6.63121_Tm_25.79 | AAGAAGAACATGA |
| HIV_all_2016LANL_WestAfricanGroupM_3571seqs_14190_F_GC_0.15_avg_30.1945_std_6.63121_Tm_19.48 | ATTCTAAAATGTA |
| HIV_all_2016LANL_WestAfricanGroupM_3571seqs_14095_F_GC_0.54_avg_30.1945_std_6.63121_Tm_35.25 | ACTCACCCTTGTC |
| HIV_all_2016LANL_WestAfricanGroupM_3571seqs_14001_F_GC_0.62_avg_30.1945_std_6.63121_Tm_38.41 | AGGACCAGCAGGA |
| HIV_all_2016LANL_WestAfricanGroupM_3571seqs_13909_F_GC_0.46_avg_30.1945_std_6.63121_Tm_32.10 | ACTCACCATTGTC |
| HIV_all_2016LANL_WestAfricanGroupM_3571seqs_13818_F_GC_0.38_avg_30.1945_std_6.63121_Tm_28.95 | ACTGCTTGTAACA |
| HIV_all_2016LANL_WestAfricanGroupM_3571seqs_13727_F_GC_0.31_avg_30.1945_std_6.63121_Tm_25.79 | GGGATTAAAGTAA |
| HIV_all_2016LANL_WestAfricanGroupM_3571seqs_13637_F_GC_0.69_avg_30.1945_std_6.63121_Tm_41.56 | CCAACAGCCCCAC |
| HIV_all_2016LANL_WestAfricanGroupM_3571seqs_13548_F_GC_0.62_avg_30.1945_std_6.63121_Tm_38.41 | CAGCAGCAGAAGG |
| HIV_all_2016LANL_WestAfricanGroupM_3571seqs_13464_F_GC_0.46_avg_30.1945_std_6.63121_Tm_32.10 | AGATCTTCAGACC |
| HIV_all_2016LANL_WestAfricanGroupM_3571seqs_13379_F_GC_0.23_avg_30.1945_std_6.63121_Tm_22.64 | TGTTATTGTAAAC |
| HIV_all_2016LANL_WestAfricanGroupM_3571seqs_13294_F_GC_0.31_avg_30.1945_std_6.63121_Tm_25.79 | AATAGACTGGTTA |
| HIV_all_2016LANL_WestAfricanGroupM_3571seqs_13210_F_GC_0.38_avg_30.1945_std_6.63121_Tm_28.95 | CATAGCAATTGGA |
| HIV_all_2016LANL_WestAfricanGroupM_3571seqs_13128_F_GC_0.38_avg_30.1945_std_6.63121_Tm_28.95 | GATGACTTGTATG |
| HIV_all_2016LANL_WestAfricanGroupM_3571seqs_13046_F_GC_0.46_avg_30.1945_std_6.63121_Tm_32.10 | TCACAGACAATGC |
| HIV_all_2016LANL_WestAfricanGroupM_3571seqs_12965_F_GC_0.62_avg_30.1945_std_6.63121_Tm_38.41 | TGGGACACAGCAG |
| HIV_all_2016LANL_WestAfricanGroupM_3571seqs_12885_F_GC_0.23_avg_30.1945_std_6.63121_Tm_22.64 | TACTTGATAGAAT |
| HIV_all_2016LANL_WestAfricanGroupM_3571seqs_12806_F_GC_0.38_avg_30.1945_std_6.63121_Tm_28.95 | TATCAAGAGCCAT |
| HIV_all_2016LANL_WestAfricanGroupM_3571seqs_12730_F_GC_0.54_avg_30.1945_std_6.63121_Tm_35.25 | TATTAAGCGGGGG |
| HIV_all_2016LANL_WestAfricanGroupM_3571seqs_12655_F_GC_0.38_avg_30.1945_std_6.63121_Tm_28.95 | ACATAAGACAAGC |
| HIV_all_2016LANL_WestAfricanGroupM_3571seqs_12580_F_GC_0.46_avg_30.1945_std_6.63121_Tm_32.10 | CACTGACAGCATT |
| HIV_all_2016LANL_WestAfricanGroupM_3571seqs_12506_F_GC_0.46_avg_30.1945_std_6.63121_Tm_32.10 | AGAATGTGGCAGA |
| HIV_all_2016LANL_WestAfricanGroupM_3571seqs_12433_F_GC_0.23_avg_30.1945_std_6.63121_Tm_22.64 | ATAAAAGATGGAT |
| HIV_all_2016LANL_WestAfricanGroupM_3571seqs_12361_F_GC_0.69_avg_30.1945_std_6.63121_Tm_41.56 | CAGCAGCAGAGGG |
| HIV_all_2016LANL_WestAfricanGroupM_3571seqs_12291_F_GC_0.38_avg_30.1945_std_6.63121_Tm_28.95 | TCTATTATGGGGT |
| HIV_all_2016LANL_WestAfricanGroupM_3571seqs_12219_F_GC_0.15_avg_30.1945_std_6.63121_Tm_19.48 | TATAGGTTAATAA |
| HIV_all_2016LANL_WestAfricanGroupM_3571seqs_12148_F_GC_0.46_avg_30.1945_std_6.63121_Tm_32.10 | AAGCACAAGAGGA |
| HIV_all_2016LANL_WestAfricanGroupM_3571seqs_12077_F_GC_0.54_avg_30.1945_std_6.63121_Tm_35.25 | ACTCACCTCTGTC |
| HIV_all_2016LANL_WestAfricanGroupM_3571seqs_12007_F_GC_0.38_avg_30.1945_std_6.63121_Tm_28.95 | AGGGGATATAAGA |
| HIV_all_2016LANL_WestAfricanGroupM_3571seqs_11937_F_GC_0.54_avg_30.1945_std_6.63121_Tm_35.25 | AGGGGGAGATATG |
| HIV_all_2016LANL_WestAfricanGroupM_3571seqs_11869_F_GC_0.31_avg_30.1945_std_6.63121_Tm_25.79 | CAAGAAAAGAATG |
| HIV_all_2016LANL_WestAfricanGroupM_3571seqs_11801_F_GC_0.62_avg_30.1945_std_6.63121_Tm_38.41 | GGGGGACATCAAG |
| HIV_all_2016LANL_WestAfricanGroupM_3571seqs_11734_F_GC_0.23_avg_30.1945_std_6.63121_Tm_22.64 | ATATGTGGAAAAA |
| HIV_all_2016LANL_WestAfricanGroupM_3571seqs_11667_F_GC_0.62_avg_30.1945_std_6.63121_Tm_38.41 | TGTGCTCCAGCTG |
| HIV_all_2016LANL_WestAfricanGroupM_3571seqs_11602_F_GC_0.23_avg_30.1945_std_6.63121_Tm_22.64 | TTGGAAGAAATAT |
| HIV_all_2016LANL_WestAfricanGroupM_3571seqs_11538_F_GC_0.62_avg_30.1945_std_6.63121_Tm_38.41 | CAGCAGATGGGGT |
| HIV_all_2016LANL_WestAfricanGroupM_3571seqs_11475_F_GC_0.54_avg_30.1945_std_6.63121_Tm_35.25 | CTCTCAAGACAGG |
| HIV_all_2016LANL_WestAfricanGroupM_3571seqs_11413_F_GC_0.62_avg_30.1945_std_6.63121_Tm_38.41 | TGGGGTTGCTCTG |
| HIV_all_2016LANL_WestAfricanGroupM_3571seqs_11289_F_GC_0.54_avg_30.1945_std_6.63121_Tm_35.25 | GTCAGCCTAAGAC |
| HIV_all_2016LANL_WestAfricanGroupM_3571seqs_11229_F_GC_0.54_avg_30.1945_std_6.63121_Tm_35.25 | GTACAGATGGCAG |
| HIV_all_2016LANL_WestAfricanGroupM_3571seqs_11169_F_GC_0.15_avg_30.1945_std_6.63121_Tm_19.48 | ATAGGAAATATAA |
| HIV_all_2016LANL_WestAfricanGroupM_3571seqs_11109_F_GC_0.38_avg_30.1945_std_6.63121_Tm_28.95 | ACTCACCATTATC |
| HIV_all_2016LANL_WestAfricanGroupM_3571seqs_11050_F_GC_0.54_avg_30.1945_std_6.63121_Tm_35.25 | GTTGGAAGTGTGG |
| HIV_all_2016LANL_WestAfricanGroupM_3571seqs_10992_F_GC_0.38_avg_30.1945_std_6.63121_Tm_28.95 | AAGTTCCAGAGAA |
| HIV_all_2016LANL_WestAfricanGroupM_3571seqs_10934_F_GC_0.23_avg_30.1945_std_6.63121_Tm_22.64 | TTTAATACAGTAG |
| HIV_all_2016LANL_WestAfricanGroupM_3571seqs_10878_F_GC_0.38_avg_30.1945_std_6.63121_Tm_28.95 | ATCATTCAAGCAC |
| HIV_all_2016LANL_WestAfricanGroupM_3571seqs_10822_F_GC_0.38_avg_30.1945_std_6.63121_Tm_28.95 | CAGGAACAAAATG |
| HIV_all_2016LANL_WestAfricanGroupM_3571seqs_10767_F_GC_0.54_avg_30.1945_std_6.63121_Tm_35.25 | TTTGGGACAGACC |
| HIV_all_2016LANL_WestAfricanGroupM_3571seqs_10713_F_GC_0.31_avg_30.1945_std_6.63121_Tm_25.79 | TAATAGTACACCT |
| HIV_all_2016LANL_WestAfricanGroupM_3571seqs_10659_F_GC_0.31_avg_30.1945_std_6.63121_Tm_25.79 | ATAACAGGAGATA |
| HIV_all_2016LANL_WestAfricanGroupM_3571seqs_10605_F_GC_0.46_avg_30.1945_std_6.63121_Tm_32.10 | CAGAAGTTATCCC |
| HIV_all_2016LANL_WestAfricanGroupM_3571seqs_10552_F_GC_0.31_avg_30.1945_std_6.63121_Tm_25.79 | GATCATCAAAATC |
| HIV_all_2016LANL_WestAfricanGroupM_3571seqs_10499_F_GC_0.46_avg_30.1945_std_6.63121_Tm_32.10 | TCTACTATGGGGT |
| HIV_all_2016LANL_WestAfricanGroupM_3571seqs_10447_F_GC_0.46_avg_30.1945_std_6.63121_Tm_32.10 | CCTATAGTGCAGA |
| HIV_all_2016LANL_WestAfricanGroupM_3571seqs_10395_F_GC_0.46_avg_30.1945_std_6.63121_Tm_32.10 | TATGGAGTACCTG |
| HIV_all_2016LANL_WestAfricanGroupM_3571seqs_10291_F_GC_0.46_avg_30.1945_std_6.63121_Tm_32.10 | CAGGAGAAAAGGA |
| HIV_all_2016LANL_WestAfricanGroupM_3571seqs_10239_F_GC_0.46_avg_30.1945_std_6.63121_Tm_32.10 | TGGGAGTTTGTCA |
| HIV_all_2016LANL_WestAfricanGroupM_3571seqs_10188_F_GC_0.62_avg_30.1945_std_6.63121_Tm_38.41 | CACCCTTGCCTAG |
| HIV_all_2016LANL_WestAfricanGroupM_3571seqs_10137_F_GC_0.31_avg_30.1945_std_6.63121_Tm_25.79 | TGCTATTGCAAAA |
| HIV_all_2016LANL_WestAfricanGroupM_3571seqs_10086_F_GC_0.31_avg_30.1945_std_6.63121_Tm_25.79 | AGGAAATTGGTAA |
| HIV_all_2016LANL_WestAfricanGroupM_3571seqs_10036_F_GC_0.23_avg_30.1945_std_6.63121_Tm_22.64 | TAATAGTACAGTT |
| HIV_all_2016LANL_WestAfricanGroupM_3571seqs_9987_F_GC_0.69_avg_30.1945_std_6.63121_Tm_41.56 | CTGAGCCAGCAGC |
| HIV_all_2016LANL_WestAfricanGroupM_3571seqs_9938_F_GC_0.31_avg_30.1945_std_6.63121_Tm_25.79 | GAAAATGGATAGT |
| HIV_all_2016LANL_WestAfricanGroupM_3571seqs_9889_F_GC_0.69_avg_30.1945_std_6.63121_Tm_41.56 | GGGATGGGGGAAG |
| HIV_all_2016LANL_WestAfricanGroupM_3571seqs_9841_F_GC_0.62_avg_30.1945_std_6.63121_Tm_38.41 | TGTGCTCCAGCAG |
| HIV_all_2016LANL_WestAfricanGroupM_3571seqs_9793_F_GC_0.46_avg_30.1945_std_6.63121_Tm_32.10 | CAATGGACATACC |
| HIV_all_2016LANL_WestAfricanGroupM_3571seqs_9746_F_GC_0.15_avg_30.1945_std_6.63121_Tm_19.48 | ATGAAAAATATCA |
| HIV_all_2016LANL_WestAfricanGroupM_3571seqs_9699_F_GC_0.31_avg_30.1945_std_6.63121_Tm_25.79 | CTGAAAATCCTTA |
| HIV_all_2016LANL_WestAfricanGroupM_3571seqs_9652_F_GC_0.15_avg_30.1945_std_6.63121_Tm_19.48 | ATGATTTATATGT |
| HIV_all_2016LANL_WestAfricanGroupM_3571seqs_9605_F_GC_0.38_avg_30.1945_std_6.63121_Tm_28.95 | TATGGAGACACTT |
| HIV_all_2016LANL_WestAfricanGroupM_3571seqs_9559_F_GC_0.23_avg_30.1945_std_6.63121_Tm_22.64 | GAGTTTTTCTATT |
| HIV_all_2016LANL_WestAfricanGroupM_3571seqs_9513_F_GC_0.62_avg_30.1945_std_6.63121_Tm_38.41 | AAACCTCCGACGC |
| HIV_all_2016LANL_WestAfricanGroupM_3571seqs_9467_F_GC_0.31_avg_30.1945_std_6.63121_Tm_25.79 | GGAGAAAAATGAA |
| HIV_all_2016LANL_WestAfricanGroupM_3571seqs_9421_F_GC_0.54_avg_30.1945_std_6.63121_Tm_35.25 | GAGAGCTTCAGGT |
| HIV_all_2016LANL_WestAfricanGroupM_3571seqs_9376_F_GC_0.62_avg_30.1945_std_6.63121_Tm_38.41 | TCGTTGCAGACCC |
| HIV_all_2016LANL_WestAfricanGroupM_3571seqs_9332_F_GC_0.23_avg_30.1945_std_6.63121_Tm_22.64 | ATAATAGTACAAC |
| HIV_all_2016LANL_WestAfricanGroupM_3571seqs_9288_F_GC_0.38_avg_30.1945_std_6.63121_Tm_28.95 | ACTAGCAGCATTA |
| HIV_all_2016LANL_WestAfricanGroupM_3571seqs_9244_F_GC_0.69_avg_30.1945_std_6.63121_Tm_41.56 | GGAGTGGGAGGAC |
| HIV_all_2016LANL_WestAfricanGroupM_3571seqs_9200_F_GC_0.38_avg_30.1945_std_6.63121_Tm_28.95 | TAATAGTGCAGCT |
| HIV_all_2016LANL_WestAfricanGroupM_3571seqs_9157_F_GC_0.46_avg_30.1945_std_6.63121_Tm_32.10 | TTGGGTTCTTAGG |
| HIV_all_2016LANL_WestAfricanGroupM_3571seqs_9114_F_GC_0.54_avg_30.1945_std_6.63121_Tm_35.25 | CTCTTCAGACAGG |
| HIV_all_2016LANL_WestAfricanGroupM_3571seqs_9072_F_GC_0.54_avg_30.1945_std_6.63121_Tm_35.25 | AATGAGACCCCAG |
| HIV_all_2016LANL_WestAfricanGroupM_3571seqs_9030_F_GC_0.62_avg_30.1945_std_6.63121_Tm_38.41 | CAGCAGCAACAGG |
| HIV_all_2016LANL_WestAfricanGroupM_3571seqs_8988_F_GC_0.38_avg_30.1945_std_6.63121_Tm_28.95 | CCATACAATACTC |
| HIV_all_2016LANL_WestAfricanGroupM_3571seqs_8946_F_GC_0.23_avg_30.1945_std_6.63121_Tm_22.64 | AAAATTACATGGT |
| HIV_all_2016LANL_WestAfricanGroupM_3571seqs_8905_F_GC_0.46_avg_30.1945_std_6.63121_Tm_32.10 | GAAACCTTCAGAC |
| HIV_all_2016LANL_WestAfricanGroupM_3571seqs_8865_F_GC_0.38_avg_30.1945_std_6.63121_Tm_28.95 | TCAGACTCATCAA |
| HIV_all_2016LANL_WestAfricanGroupM_3571seqs_8825_F_GC_0.54_avg_30.1945_std_6.63121_Tm_35.25 | TTTGGCAACGACC |
| HIV_all_2016LANL_WestAfricanGroupM_3571seqs_8785_F_GC_0.46_avg_30.1945_std_6.63121_Tm_32.10 | AATCGCAGAACCA |
| HIV_all_2016LANL_WestAfricanGroupM_3571seqs_8745_F_GC_0.31_avg_30.1945_std_6.63121_Tm_25.79 | ACTGCTTGTAATA |
| HIV_all_2016LANL_WestAfricanGroupM_3571seqs_8705_F_GC_0.38_avg_30.1945_std_6.63121_Tm_28.95 | TGCAGAATAAAGC |
| HIV_all_2016LANL_WestAfricanGroupM_3571seqs_8665_F_GC_0.46_avg_30.1945_std_6.63121_Tm_32.10 | TCAGCAACTCCTA |
| HIV_all_2016LANL_WestAfricanGroupM_3571seqs_8626_F_GC_0.62_avg_30.1945_std_6.63121_Tm_38.41 | CTCTCGACGCAAG |
| HIV_all_2016LANL_WestAfricanGroupM_3571seqs_8549_F_GC_0.38_avg_30.1945_std_6.63121_Tm_28.95 | TATTACAGGGACA |
| HIV_all_2016LANL_WestAfricanGroupM_3571seqs_8511_F_GC_0.54_avg_30.1945_std_6.63121_Tm_35.25 | CCATAGGAGGACA |
| HIV_all_2016LANL_WestAfricanGroupM_3571seqs_8474_F_GC_0.62_avg_30.1945_std_6.63121_Tm_38.41 | AGATGGGTGCGAG |
| HIV_all_2016LANL_WestAfricanGroupM_3571seqs_8437_F_GC_0.31_avg_30.1945_std_6.63121_Tm_25.79 | ACTCCTTGTAATA |
| HIV_all_2016LANL_WestAfricanGroupM_3571seqs_8400_F_GC_0.54_avg_30.1945_std_6.63121_Tm_35.25 | TTCAGACCTGCAG |
| HIV_all_2016LANL_WestAfricanGroupM_3571seqs_8363_F_GC_0.54_avg_30.1945_std_6.63121_Tm_35.25 | ATCAACCCCTCTC |
| HIV_all_2016LANL_WestAfricanGroupM_3571seqs_8289_F_GC_0.23_avg_30.1945_std_6.63121_Tm_22.64 | TTGTAACATTAGT |
| HIV_all_2016LANL_WestAfricanGroupM_3571seqs_8253_F_GC_0.62_avg_30.1945_std_6.63121_Tm_38.41 | CCGCCTTTGCCTA |
| HIV_all_2016LANL_WestAfricanGroupM_3571seqs_8217_F_GC_0.62_avg_30.1945_std_6.63121_Tm_38.41 | GGCTTCCCAGTCA |
| HIV_all_2016LANL_WestAfricanGroupM_3571seqs_8181_F_GC_0.46_avg_30.1945_std_6.63121_Tm_32.10 | ACAGGAAACAGCA |
| HIV_all_2016LANL_WestAfricanGroupM_3571seqs_8146_F_GC_0.54_avg_30.1945_std_6.63121_Tm_35.25 | GCAGGTAGCACTA |
| HIV_all_2016LANL_WestAfricanGroupM_3571seqs_8111_F_GC_0.23_avg_30.1945_std_6.63121_Tm_22.64 | TAATTGGAAGAAA |
| HIV_all_2016LANL_WestAfricanGroupM_3571seqs_8076_F_GC_0.69_avg_30.1945_std_6.63121_Tm_41.56 | CTCCCCTACCCAG |
| HIV_all_2016LANL_WestAfricanGroupM_3571seqs_8041_F_GC_0.38_avg_30.1945_std_6.63121_Tm_28.95 | GTCAAAATTACCC |
| HIV_all_2016LANL_WestAfricanGroupM_3571seqs_8006_F_GC_0.54_avg_30.1945_std_6.63121_Tm_35.25 | ATGCCAGGGGAAA |
| HIV_all_2016LANL_WestAfricanGroupM_3571seqs_7972_F_GC_0.31_avg_30.1945_std_6.63121_Tm_25.79 | AAAACTGCTCTTT |
| HIV_all_2016LANL_WestAfricanGroupM_3571seqs_7939_F_GC_0.54_avg_30.1945_std_6.63121_Tm_35.25 | CAGAAGGAGTAGG |
| HIV_all_2016LANL_WestAfricanGroupM_3571seqs_7907_F_GC_0.23_avg_30.1945_std_6.63121_Tm_22.64 | AATATACTGCATT |
| HIV_all_2016LANL_WestAfricanGroupM_3571seqs_7875_F_GC_0.46_avg_30.1945_std_6.63121_Tm_32.10 | TGGGTTTTCCAGT |
| HIV_all_2016LANL_WestAfricanGroupM_3571seqs_7843_F_GC_0.38_avg_30.1945_std_6.63121_Tm_28.95 | CTCAGTCATTACA |
| HIV_all_2016LANL_WestAfricanGroupM_3571seqs_7811_F_GC_0.54_avg_30.1945_std_6.63121_Tm_35.25 | CCAGTAAGACCTC |
| HIV_all_2016LANL_WestAfricanGroupM_3571seqs_7779_F_GC_0.23_avg_30.1945_std_6.63121_Tm_22.64 | TTAAGACAAAGAA |
| HIV_all_2016LANL_WestAfricanGroupM_3571seqs_7747_F_GC_0.15_avg_30.1945_std_6.63121_Tm_19.48 | AAAAGAAAATAGA |
| HIV_all_2016LANL_WestAfricanGroupM_3571seqs_7715_F_GC_0.54_avg_30.1945_std_6.63121_Tm_35.25 | GCTGGAAATGTGG |
| HIV_all_2016LANL_WestAfricanGroupM_3571seqs_7684_F_GC_0.38_avg_30.1945_std_6.63121_Tm_28.95 | CAGGAGAAAAAGA |
| HIV_all_2016LANL_WestAfricanGroupM_3571seqs_7653_F_GC_0.54_avg_30.1945_std_6.63121_Tm_35.25 | AAGGGGTTGAAGC |
| HIV_all_2016LANL_WestAfricanGroupM_3571seqs_7622_F_GC_0.62_avg_30.1945_std_6.63121_Tm_38.41 | CAGACCCTTACCC |
| HIV_all_2016LANL_WestAfricanGroupM_3571seqs_7593_F_GC_0.31_avg_30.1945_std_6.63121_Tm_25.79 | TATATCAGGATGA |
| HIV_all_2016LANL_WestAfricanGroupM_3571seqs_7563_F_GC_0.38_avg_30.1945_std_6.63121_Tm_28.95 | TATGCAACAGTCT |
| HIV_all_2016LANL_WestAfricanGroupM_3571seqs_7533_F_GC_0.38_avg_30.1945_std_6.63121_Tm_28.95 | CAGGAAAAGAATG |
| HIV_all_2016LANL_WestAfricanGroupM_3571seqs_7503_F_GC_0.69_avg_30.1945_std_6.63121_Tm_41.56 | CTCCTGCTCCAGC |
| HIV_all_2016LANL_WestAfricanGroupM_3571seqs_7475_F_GC_0.31_avg_30.1945_std_6.63121_Tm_25.79 | TTTATTATGGGGT |
| HIV_all_2016LANL_WestAfricanGroupM_3571seqs_7445_F_GC_0.15_avg_30.1945_std_6.63121_Tm_19.48 | TAATAAACTGTAA |
| HIV_all_2016LANL_WestAfricanGroupM_3571seqs_7415_F_GC_0.38_avg_30.1945_std_6.63121_Tm_28.95 | AGAAGAGATGATG |
| HIV_all_2016LANL_WestAfricanGroupM_3571seqs_7355_F_GC_0.54_avg_30.1945_std_6.63121_Tm_35.25 | GCAGTGCAAATGG |
| HIV_all_2016LANL_WestAfricanGroupM_3571seqs_7326_F_GC_0.38_avg_30.1945_std_6.63121_Tm_28.95 | TGGGAATTTGTCA |
| HIV_all_2016LANL_WestAfricanGroupM_3571seqs_7297_F_GC_0.54_avg_30.1945_std_6.63121_Tm_35.25 | TCAGGGGACTACA |
| HIV_all_2016LANL_WestAfricanGroupM_3571seqs_7268_F_GC_0.23_avg_30.1945_std_6.63121_Tm_22.64 | AGAATAAGACAAA |
| HIV_all_2016LANL_WestAfricanGroupM_3571seqs_7239_F_GC_0.46_avg_30.1945_std_6.63121_Tm_32.10 | GTAAGGTCATGGA |
| HIV_all_2016LANL_WestAfricanGroupM_3571seqs_7182_F_GC_0.46_avg_30.1945_std_6.63121_Tm_32.10 | AGACATTCAGACC |
| HIV_all_2016LANL_WestAfricanGroupM_3571seqs_7155_F_GC_0.15_avg_30.1945_std_6.63121_Tm_19.48 | AATTCTTTTATTG |
| HIV_all_2016LANL_WestAfricanGroupM_3571seqs_7127_F_GC_0.54_avg_30.1945_std_6.63121_Tm_35.25 | TGCACCTGGAGTA |
| HIV_all_2016LANL_WestAfricanGroupM_3571seqs_7099_F_GC_0.69_avg_30.1945_std_6.63121_Tm_41.56 | CACCCCTGCCTAG |
| HIV_all_2016LANL_WestAfricanGroupM_3571seqs_7071_F_GC_0.54_avg_30.1945_std_6.63121_Tm_35.25 | AAGGGACTGAGAC |
| HIV_all_2016LANL_WestAfricanGroupM_3571seqs_7043_F_GC_0.38_avg_30.1945_std_6.63121_Tm_28.95 | AGGAACAGAAGAA |
| HIV_all_2016LANL_WestAfricanGroupM_3571seqs_7016_F_GC_0.38_avg_30.1945_std_6.63121_Tm_28.95 | AGGCACATTGTAA |
| HIV_all_2016LANL_WestAfricanGroupM_3571seqs_6989_F_GC_0.54_avg_30.1945_std_6.63121_Tm_35.25 | GACTTGGACTGTG |
| HIV_all_2016LANL_WestAfricanGroupM_3571seqs_6962_F_GC_0.31_avg_30.1945_std_6.63121_Tm_25.79 | TGGGAATTTGTTA |
| HIV_all_2016LANL_WestAfricanGroupM_3571seqs_6935_F_GC_0.54_avg_30.1945_std_6.63121_Tm_35.25 | ATTGGGCCAGTCA |
| HIV_all_2016LANL_WestAfricanGroupM_3571seqs_6883_F_GC_0.23_avg_30.1945_std_6.63121_Tm_22.64 | TGTAATGATAAGA |
| HIV_all_2016LANL_WestAfricanGroupM_3571seqs_6857_F_GC_0.62_avg_30.1945_std_6.63121_Tm_38.41 | AGCCACCTTTGCC |
| HIV_all_2016LANL_WestAfricanGroupM_3571seqs_6831_F_GC_0.31_avg_30.1945_std_6.63121_Tm_25.79 | CATGAGAAATATC |
| HIV_all_2016LANL_WestAfricanGroupM_3571seqs_6805_F_GC_0.23_avg_30.1945_std_6.63121_Tm_22.64 | AACAATGCTAAAA |
| HIV_all_2016LANL_WestAfricanGroupM_3571seqs_6779_F_GC_0.31_avg_30.1945_std_6.63121_Tm_25.79 | TACTTACAGAATC |
| HIV_all_2016LANL_WestAfricanGroupM_3571seqs_6753_F_GC_0.54_avg_30.1945_std_6.63121_Tm_35.25 | ACAGGATCAGAGG |
| HIV_all_2016LANL_WestAfricanGroupM_3571seqs_6728_F_GC_0.69_avg_30.1945_std_6.63121_Tm_41.56 | GGACACCAGGCAG |
| HIV_all_2016LANL_WestAfricanGroupM_3571seqs_6703_F_GC_0.38_avg_30.1945_std_6.63121_Tm_28.95 | AACTGCTTGCAAT |
| HIV_all_2016LANL_WestAfricanGroupM_3571seqs_6653_F_GC_0.31_avg_30.1945_std_6.63121_Tm_25.79 | AACAATGCCAAAA |
| HIV_all_2016LANL_WestAfricanGroupM_3571seqs_6628_F_GC_0.62_avg_30.1945_std_6.63121_Tm_38.41 | TCCTCCAGCAGCA |
| HIV_all_2016LANL_WestAfricanGroupM_3571seqs_6603_F_GC_0.38_avg_30.1945_std_6.63121_Tm_28.95 | ACTCACCTTTATC |
| HIV_all_2016LANL_WestAfricanGroupM_3571seqs_6578_F_GC_0.54_avg_30.1945_std_6.63121_Tm_35.25 | AGCGAGGATTGTG |
| HIV_all_2016LANL_WestAfricanGroupM_3571seqs_6553_F_GC_0.31_avg_30.1945_std_6.63121_Tm_25.79 | TTGAAAAATCGCA |
| HIV_all_2016LANL_WestAfricanGroupM_3571seqs_6505_F_GC_0.31_avg_30.1945_std_6.63121_Tm_25.79 | ATATGTTGACTCA |
| HIV_all_2016LANL_WestAfricanGroupM_3571seqs_6481_F_GC_0.46_avg_30.1945_std_6.63121_Tm_32.10 | TAGGGGACATAAG |
| HIV_all_2016LANL_WestAfricanGroupM_3571seqs_6458_F_GC_0.54_avg_30.1945_std_6.63121_Tm_35.25 | ACTCACCTGTGTC |
| HIV_all_2016LANL_WestAfricanGroupM_3571seqs_6435_F_GC_0.38_avg_30.1945_std_6.63121_Tm_28.95 | GATCTGACTTAGA |
| HIV_all_2016LANL_WestAfricanGroupM_3571seqs_6412_F_GC_0.46_avg_30.1945_std_6.63121_Tm_32.10 | AGATTTACCCAGG |
| HIV_all_2016LANL_WestAfricanGroupM_3571seqs_6389_F_GC_0.62_avg_30.1945_std_6.63121_Tm_38.41 | AGCGAGGACTGTG |
| HIV_all_2016LANL_WestAfricanGroupM_3571seqs_6367_F_GC_0.38_avg_30.1945_std_6.63121_Tm_28.95 | TTGCAATACCTCA |
| HIV_all_2016LANL_WestAfricanGroupM_3571seqs_6345_F_GC_0.62_avg_30.1945_std_6.63121_Tm_38.41 | CAACAGCTCCACC |
| HIV_all_2016LANL_WestAfricanGroupM_3571seqs_6323_F_GC_0.38_avg_30.1945_std_6.63121_Tm_28.95 | CAAAACCAACAAG |
| HIV_all_2016LANL_WestAfricanGroupM_3571seqs_6301_F_GC_0.69_avg_30.1945_std_6.63121_Tm_41.56 | GGCCAGGGGGAAA |
| HIV_all_2016LANL_WestAfricanGroupM_3571seqs_6279_F_GC_0.31_avg_30.1945_std_6.63121_Tm_25.79 | TAGAACAAATGCA |
| HIV_all_2016LANL_WestAfricanGroupM_3571seqs_6257_F_GC_0.23_avg_30.1945_std_6.63121_Tm_22.64 | ATGAAAGATATCA |
| HIV_all_2016LANL_WestAfricanGroupM_3571seqs_6235_F_GC_0.31_avg_30.1945_std_6.63121_Tm_25.79 | TGCTATTGTAAAG |
| HIV_all_2016LANL_WestAfricanGroupM_3571seqs_6213_F_GC_0.38_avg_30.1945_std_6.63121_Tm_28.95 | TCACTATACCTAG |
| HIV_all_2016LANL_WestAfricanGroupM_3571seqs_6191_F_GC_0.62_avg_30.1945_std_6.63121_Tm_38.41 | GGACATCAGGCAG |
| HIV_all_2016LANL_WestAfricanGroupM_3571seqs_6169_F_GC_0.38_avg_30.1945_std_6.63121_Tm_28.95 | AACCAGCAAGAAA |
| HIV_all_2016LANL_WestAfricanGroupM_3571seqs_6147_F_GC_0.38_avg_30.1945_std_6.63121_Tm_28.95 | GAGATATTCAGAC |
| HIV_all_2016LANL_WestAfricanGroupM_3571seqs_6125_F_GC_0.15_avg_30.1945_std_6.63121_Tm_19.48 | ATAAAACCAAAAA |
| HIV_all_2016LANL_WestAfricanGroupM_3571seqs_6104_F_GC_0.15_avg_30.1945_std_6.63121_Tm_19.48 | AATAAATAGGTTA |
| HIV_all_2016LANL_WestAfricanGroupM_3571seqs_6083_F_GC_0.46_avg_30.1945_std_6.63121_Tm_32.10 | TAAGACAAACCCC |
| HIV_all_2016LANL_WestAfricanGroupM_3571seqs_6062_F_GC_0.54_avg_30.1945_std_6.63121_Tm_35.25 | CAGGAGTTGAAGC |
| HIV_all_2016LANL_WestAfricanGroupM_3571seqs_6041_F_GC_0.62_avg_30.1945_std_6.63121_Tm_38.41 | CAGCAGGAAGCAC |
| HIV_all_2016LANL_WestAfricanGroupM_3571seqs_6020_F_GC_0.15_avg_30.1945_std_6.63121_Tm_19.48 | TGATAAATTGTAA |
| HIV_all_2016LANL_WestAfricanGroupM_3571seqs_5999_F_GC_0.23_avg_30.1945_std_6.63121_Tm_22.64 | GGAGAAATAAAAA |
| HIV_all_2016LANL_WestAfricanGroupM_3571seqs_5978_F_GC_0.38_avg_30.1945_std_6.63121_Tm_28.95 | ATGGAGAGTTCTT |
| HIV_all_2016LANL_WestAfricanGroupM_3571seqs_5958_F_GC_0.31_avg_30.1945_std_6.63121_Tm_25.79 | ATTGTAATGCACA |
| HIV_all_2016LANL_WestAfricanGroupM_3571seqs_5938_F_GC_0.38_avg_30.1945_std_6.63121_Tm_28.95 | TAGTGACCCTAAA |
| HIV_all_2016LANL_WestAfricanGroupM_3571seqs_5919_F_GC_0.54_avg_30.1945_std_6.63121_Tm_35.25 | CACCCATGTTGCT |
| HIV_all_2016LANL_WestAfricanGroupM_3571seqs_5900_F_GC_0.38_avg_30.1945_std_6.63121_Tm_28.95 | TGGACATATCAGA |
| HIV_all_2016LANL_WestAfricanGroupM_3571seqs_5881_F_GC_0.38_avg_30.1945_std_6.63121_Tm_28.95 | GGGGAAAATTAGA |
| HIV_all_2016LANL_WestAfricanGroupM_3571seqs_5862_F_GC_0.23_avg_30.1945_std_6.63121_Tm_22.64 | AAAATTGCTCTTT |
| HIV_all_2016LANL_WestAfricanGroupM_3571seqs_5843_F_GC_0.46_avg_30.1945_std_6.63121_Tm_32.10 | GAAACAGGACAAG |
| HIV_all_2016LANL_WestAfricanGroupM_3571seqs_5824_F_GC_0.38_avg_30.1945_std_6.63121_Tm_28.95 | CATATAACACTCC |
| HIV_all_2016LANL_WestAfricanGroupM_3571seqs_5805_F_GC_0.62_avg_30.1945_std_6.63121_Tm_38.41 | CCCTTCAGACAGG |
| HIV_all_2016LANL_WestAfricanGroupM_3571seqs_5786_F_GC_0.38_avg_30.1945_std_6.63121_Tm_28.95 | TAATAGTGCACCT |
| HIV_all_2016LANL_WestAfricanGroupM_3571seqs_5768_F_GC_0.23_avg_30.1945_std_6.63121_Tm_22.64 | ATGCAGAATAAAA |
| HIV_all_2016LANL_WestAfricanGroupM_3571seqs_5750_F_GC_0.31_avg_30.1945_std_6.63121_Tm_25.79 | TCAAAACAGTAAG |
| HIV_all_2016LANL_WestAfricanGroupM_3571seqs_5732_F_GC_0.23_avg_30.1945_std_6.63121_Tm_22.64 | AAGCATATTGTAA |
| HIV_all_2016LANL_WestAfricanGroupM_3571seqs_5714_F_GC_0.62_avg_30.1945_std_6.63121_Tm_38.41 | CAGGAGTGGAAGC |
| HIV_all_2016LANL_WestAfricanGroupM_3571seqs_5696_F_GC_0.54_avg_30.1945_std_6.63121_Tm_35.25 | TATGGGCAAGCAG |
| HIV_all_2016LANL_WestAfricanGroupM_3571seqs_5678_F_GC_0.62_avg_30.1945_std_6.63121_Tm_38.41 | CTGAACCAGCAGC |
| HIV_all_2016LANL_WestAfricanGroupM_3571seqs_5660_F_GC_0.69_avg_30.1945_std_6.63121_Tm_41.56 | CCCCACCAGCAGA |
| HIV_all_2016LANL_WestAfricanGroupM_3571seqs_5642_F_GC_0.23_avg_30.1945_std_6.63121_Tm_22.64 | AATAAGACATTCA |
| HIV_all_2016LANL_WestAfricanGroupM_3571seqs_5625_F_GC_0.69_avg_30.1945_std_6.63121_Tm_41.56 | CAGACCCCTACCC |
| HIV_all_2016LANL_WestAfricanGroupM_3571seqs_5608_F_GC_0.46_avg_30.1945_std_6.63121_Tm_32.10 | CAGATGCATGAAG |
| HIV_all_2016LANL_WestAfricanGroupM_3571seqs_5591_F_GC_0.69_avg_30.1945_std_6.63121_Tm_41.56 | CCCTCCAGCAGCA |
| HIV_all_2016LANL_WestAfricanGroupM_3571seqs_5574_F_GC_0.46_avg_30.1945_std_6.63121_Tm_32.10 | AACTGCTTGTAGC |
| HIV_all_2016LANL_WestAfricanGroupM_3571seqs_5557_F_GC_0.69_avg_30.1945_std_6.63121_Tm_41.56 | GAGCCAACAGCCC |
| HIV_all_2016LANL_WestAfricanGroupM_3571seqs_5540_F_GC_0.15_avg_30.1945_std_6.63121_Tm_19.48 | GATAAAACAAATT |
| HIV_all_2016LANL_WestAfricanGroupM_3571seqs_5523_F_GC_0.54_avg_30.1945_std_6.63121_Tm_35.25 | GGAGGAGACATGA |
| HIV_all_2016LANL_WestAfricanGroupM_3571seqs_5506_F_GC_0.31_avg_30.1945_std_6.63121_Tm_25.79 | AAGCATATTGTGA |
| HIV_all_2016LANL_WestAfricanGroupM_3571seqs_5489_F_GC_0.31_avg_30.1945_std_6.63121_Tm_25.79 | TTACAACACATAG |
| HIV_all_2016LANL_WestAfricanGroupM_3571seqs_5472_F_GC_0.23_avg_30.1945_std_6.63121_Tm_22.64 | AATAGATAGGTTA |
| HIV_all_2016LANL_WestAfricanGroupM_3571seqs_5455_F_GC_0.62_avg_30.1945_std_6.63121_Tm_38.41 | GTGGGCTTTCCAG |
| HIV_all_2016LANL_WestAfricanGroupM_3571seqs_5439_F_GC_0.31_avg_30.1945_std_6.63121_Tm_25.79 | AGAAGTTATTCCA |
| HIV_all_2016LANL_WestAfricanGroupM_3571seqs_5423_F_GC_0.31_avg_30.1945_std_6.63121_Tm_25.79 | TTGAAGAATCACA |
| HIV_all_2016LANL_WestAfricanGroupM_3571seqs_5407_F_GC_0.46_avg_30.1945_std_6.63121_Tm_32.10 | AAATCCAGCAGGA |
| HIV_all_2016LANL_WestAfricanGroupM_3571seqs_5391_F_GC_0.46_avg_30.1945_std_6.63121_Tm_32.10 | AGATACCTACAGG |
| HIV_all_2016LANL_WestAfricanGroupM_3571seqs_5375_F_GC_0.46_avg_30.1945_std_6.63121_Tm_32.10 | GGTCACAGTCTAT |
| HIV_all_2016LANL_WestAfricanGroupM_3571seqs_5359_F_GC_0.23_avg_30.1945_std_6.63121_Tm_22.64 | CCTGTTAATATTA |
| HIV_all_2016LANL_WestAfricanGroupM_3571seqs_5343_F_GC_0.38_avg_30.1945_std_6.63121_Tm_28.95 | GGACAACATATCT |
| HIV_all_2016LANL_WestAfricanGroupM_3571seqs_5327_F_GC_0.38_avg_30.1945_std_6.63121_Tm_28.95 | AAGTTGTAACACC |
| HIV_all_2016LANL_WestAfricanGroupM_3571seqs_5311_F_GC_0.31_avg_30.1945_std_6.63121_Tm_25.79 | CACTAAAAGCATT |
| HIV_all_2016LANL_WestAfricanGroupM_3571seqs_5295_F_GC_0.62_avg_30.1945_std_6.63121_Tm_38.41 | TTTGGCAGCGACC |
| HIV_all_2016LANL_WestAfricanGroupM_3571seqs_5279_F_GC_0.62_avg_30.1945_std_6.63121_Tm_38.41 | CAAACCCAGCAGG |
| HIV_all_2016LANL_WestAfricanGroupM_3571seqs_5263_F_GC_0.46_avg_30.1945_std_6.63121_Tm_32.10 | ACTCCTTGTACCA |
| HIV_all_2016LANL_WestAfricanGroupM_3571seqs_5247_F_GC_0.62_avg_30.1945_std_6.63121_Tm_38.41 | TCCTGCAGCAGCA |
| HIV_all_2016LANL_WestAfricanGroupM_3571seqs_5232_F_GC_0.54_avg_30.1945_std_6.63121_Tm_35.25 | AATTGGGCGAGTC |
| HIV_all_2016LANL_WestAfricanGroupM_3571seqs_5217_F_GC_0.31_avg_30.1945_std_6.63121_Tm_25.79 | GACAATGCTAAAA |
| HIV_all_2016LANL_WestAfricanGroupM_3571seqs_5202_F_GC_0.54_avg_30.1945_std_6.63121_Tm_35.25 | ACAGGAACAGAGG |
| HIV_all_2016LANL_WestAfricanGroupM_3571seqs_5187_F_GC_0.31_avg_30.1945_std_6.63121_Tm_25.79 | AATCATCAAGATC |
| HIV_all_2016LANL_WestAfricanGroupM_3571seqs_5172_F_GC_0.38_avg_30.1945_std_6.63121_Tm_28.95 | ACATAGTGACAGA |
| HIV_all_2016LANL_WestAfricanGroupM_3571seqs_5157_F_GC_0.23_avg_30.1945_std_6.63121_Tm_22.64 | GATAAAGCAAATT |
| HIV_all_2016LANL_WestAfricanGroupM_3571seqs_5142_F_GC_0.62_avg_30.1945_std_6.63121_Tm_38.41 | GGCATCAAGCAGC |
| HIV_all_2016LANL_WestAfricanGroupM_3571seqs_5127_F_GC_0.23_avg_30.1945_std_6.63121_Tm_22.64 | ATTGAGAGAATAA |
| HIV_all_2016LANL_WestAfricanGroupM_3571seqs_5112_F_GC_0.31_avg_30.1945_std_6.63121_Tm_25.79 | CTATAGTGCAAAA |
| HIV_all_2016LANL_WestAfricanGroupM_3571seqs_5097_F_GC_0.38_avg_30.1945_std_6.63121_Tm_28.95 | ATCTGACAGACAA |
| HIV_all_2016LANL_WestAfricanGroupM_3571seqs_5082_F_GC_0.54_avg_30.1945_std_6.63121_Tm_35.25 | CTCCTCAAGACAG |
| HIV_all_2016LANL_WestAfricanGroupM_3571seqs_5067_F_GC_0.31_avg_30.1945_std_6.63121_Tm_25.79 | AGAATAAGACAAG |
| HIV_all_2016LANL_WestAfricanGroupM_3571seqs_5052_F_GC_0.15_avg_30.1945_std_6.63121_Tm_19.48 | TATAGACTAATAA |
| HIV_all_2016LANL_WestAfricanGroupM_3571seqs_5037_F_GC_0.46_avg_30.1945_std_6.63121_Tm_32.10 | GTAGGTTTTCCAG |
| HIV_all_2016LANL_WestAfricanGroupM_3571seqs_5022_F_GC_0.54_avg_30.1945_std_6.63121_Tm_35.25 | AGAGAAAGCCACC |
| HIV_all_2016LANL_WestAfricanGroupM_3571seqs_4992_F_GC_0.54_avg_30.1945_std_6.63121_Tm_35.25 | GTGGAACTTCTGG |
| HIV_all_2016LANL_WestAfricanGroupM_3571seqs_4977_F_GC_0.62_avg_30.1945_std_6.63121_Tm_38.41 | TCGTTCCAGACCC |
| HIV_all_2016LANL_WestAfricanGroupM_3571seqs_4963_F_GC_0.62_avg_30.1945_std_6.63121_Tm_38.41 | ACTCGGCTTGCTG |
| HIV_all_2016LANL_WestAfricanGroupM_3571seqs_4949_F_GC_0.31_avg_30.1945_std_6.63121_Tm_25.79 | TAGGAAACATAAG |
| HIV_all_2016LANL_WestAfricanGroupM_3571seqs_4935_F_GC_0.31_avg_30.1945_std_6.63121_Tm_25.79 | TTAGATAAATGGG |
| HIV_all_2016LANL_WestAfricanGroupM_3571seqs_4921_F_GC_0.62_avg_30.1945_std_6.63121_Tm_38.41 | GGCACTTGGGTCA |
| HIV_all_2016LANL_WestAfricanGroupM_3571seqs_4907_F_GC_0.69_avg_30.1945_std_6.63121_Tm_41.56 | AGAGGGGGTGGGA |
| HIV_all_2016LANL_WestAfricanGroupM_3571seqs_4893_F_GC_0.23_avg_30.1945_std_6.63121_Tm_22.64 | TTTTAATATGTGG |
| HIV_all_2016LANL_WestAfricanGroupM_3571seqs_4879_F_GC_0.31_avg_30.1945_std_6.63121_Tm_25.79 | TCAAAATCCTGTA |
| HIV_all_2016LANL_WestAfricanGroupM_3571seqs_4865_F_GC_0.46_avg_30.1945_std_6.63121_Tm_32.10 | AGACTTTCAGACC |
| HIV_all_2016LANL_WestAfricanGroupM_3571seqs_4851_F_GC_0.38_avg_30.1945_std_6.63121_Tm_28.95 | AAATCTTCAGACC |
| HIV_all_2016LANL_WestAfricanGroupM_3571seqs_4837_F_GC_0.23_avg_30.1945_std_6.63121_Tm_22.64 | TATAGGCTAATAA |
| HIV_all_2016LANL_WestAfricanGroupM_3571seqs_4823_F_GC_0.23_avg_30.1945_std_6.63121_Tm_22.64 | TAATACTAGCTAT |
| HIV_all_2016LANL_WestAfricanGroupM_3571seqs_4809_F_GC_0.15_avg_30.1945_std_6.63121_Tm_19.48 | AGTATAGATTAAT |
| HIV_all_2016LANL_WestAfricanGroupM_3571seqs_4795_F_GC_0.62_avg_30.1945_std_6.63121_Tm_38.41 | TATGGGGTGCCTG |
| HIV_all_2016LANL_WestAfricanGroupM_3571seqs_4781_F_GC_0.62_avg_30.1945_std_6.63121_Tm_38.41 | AGCAGAAGGGGTG |
| HIV_all_2016LANL_WestAfricanGroupM_3571seqs_4767_F_GC_0.54_avg_30.1945_std_6.63121_Tm_35.25 | AATGCACAAGGGC |
| HIV_all_2016LANL_WestAfricanGroupM_3571seqs_4753_F_GC_0.38_avg_30.1945_std_6.63121_Tm_28.95 | GAGTATCCATAGA |
| HIV_all_2016LANL_WestAfricanGroupM_3571seqs_4740_F_GC_0.31_avg_30.1945_std_6.63121_Tm_25.79 | GATGACTTATATG |
| HIV_all_2016LANL_WestAfricanGroupM_3571seqs_4727_F_GC_0.69_avg_30.1945_std_6.63121_Tm_41.56 | GGCAGGAGGTAGG |
| HIV_all_2016LANL_WestAfricanGroupM_3571seqs_4714_F_GC_0.62_avg_30.1945_std_6.63121_Tm_38.41 | CAGCAGAGGGAGT |
| HIV_all_2016LANL_WestAfricanGroupM_3571seqs_4701_F_GC_0.31_avg_30.1945_std_6.63121_Tm_25.79 | AACAGTGTACTAT |
| HIV_all_2016LANL_WestAfricanGroupM_3571seqs_4688_F_GC_0.31_avg_30.1945_std_6.63121_Tm_25.79 | ATACAGGCTAATA |
| HIV_all_2016LANL_WestAfricanGroupM_3571seqs_4675_F_GC_0.38_avg_30.1945_std_6.63121_Tm_28.95 | CAGGAAAGAAATG |
| HIV_all_2016LANL_WestAfricanGroupM_3571seqs_4662_F_GC_0.31_avg_30.1945_std_6.63121_Tm_25.79 | TCACAAATAATGC |
| HIV_all_2016LANL_WestAfricanGroupM_3571seqs_4649_F_GC_0.38_avg_30.1945_std_6.63121_Tm_28.95 | AACACCAAAAAGG |
| HIV_all_2016LANL_WestAfricanGroupM_3571seqs_4636_F_GC_0.38_avg_30.1945_std_6.63121_Tm_28.95 | ACTCCTTGTACTA |
| HIV_all_2016LANL_WestAfricanGroupM_3571seqs_4623_F_GC_0.38_avg_30.1945_std_6.63121_Tm_28.95 | AGAATTAGACAGG |
| HIV_all_2016LANL_WestAfricanGroupM_3571seqs_4610_F_GC_0.38_avg_30.1945_std_6.63121_Tm_28.95 | AGGAGACATAAGA |
| HIV_all_2016LANL_WestAfricanGroupM_3571seqs_4597_F_GC_0.69_avg_30.1945_std_6.63121_Tm_41.56 | TGCAGGGCCCCTA |
| HIV_all_2016LANL_WestAfricanGroupM_3571seqs_4584_F_GC_0.62_avg_30.1945_std_6.63121_Tm_38.41 | GAACTGAGCCAGC |
| HIV_all_2016LANL_WestAfricanGroupM_3571seqs_4571_F_GC_0.31_avg_30.1945_std_6.63121_Tm_25.79 | AAAGCGAAAGTAA |
| HIV_all_2016LANL_WestAfricanGroupM_3571seqs_4558_F_GC_0.31_avg_30.1945_std_6.63121_Tm_25.79 | CAGAAGAAAATAG |
| HIV_all_2016LANL_WestAfricanGroupM_3571seqs_4545_F_GC_0.38_avg_30.1945_std_6.63121_Tm_28.95 | ACCCCTTGTAATA |
| HIV_all_2016LANL_WestAfricanGroupM_3571seqs_4532_F_GC_0.54_avg_30.1945_std_6.63121_Tm_35.25 | AACTCCTCCAGCA |
| HIV_all_2016LANL_WestAfricanGroupM_3571seqs_4520_F_GC_0.38_avg_30.1945_std_6.63121_Tm_28.95 | GCTGAACATCTTA |
| HIV_all_2016LANL_WestAfricanGroupM_3571seqs_4507_F_GC_0.54_avg_30.1945_std_6.63121_Tm_35.25 | TCGTTACAGACCC |
| HIV_all_2016LANL_WestAfricanGroupM_3571seqs_4495_F_GC_0.31_avg_30.1945_std_6.63121_Tm_25.79 | CTGTCAACATAAT |
| HIV_all_2016LANL_WestAfricanGroupM_3571seqs_4471_F_GC_0.62_avg_30.1945_std_6.63121_Tm_38.41 | AGCAGCACCAGGA |
| HIV_all_2016LANL_WestAfricanGroupM_3571seqs_4460_F_GC_0.46_avg_30.1945_std_6.63121_Tm_32.10 | GTAACACCTCAGT |
| HIV_all_2016LANL_WestAfricanGroupM_3571seqs_4448_F_GC_0.31_avg_30.1945_std_6.63121_Tm_25.79 | TATTACAGAGACA |
| HIV_all_2016LANL_WestAfricanGroupM_3571seqs_4436_F_GC_0.23_avg_30.1945_std_6.63121_Tm_22.64 | GGAATTAAAGTAA |
| HIV_all_2016LANL_WestAfricanGroupM_3571seqs_4424_F_GC_0.38_avg_30.1945_std_6.63121_Tm_28.95 | ATAAGACAAGCAC |
| HIV_all_2016LANL_WestAfricanGroupM_3571seqs_4412_F_GC_0.31_avg_30.1945_std_6.63121_Tm_25.79 | TTGTAATGTCTCA |
| HIV_all_2016LANL_WestAfricanGroupM_3571seqs_4400_F_GC_0.54_avg_30.1945_std_6.63121_Tm_35.25 | GTAGGTTTCCCAG |
| HIV_all_2016LANL_WestAfricanGroupM_3571seqs_4388_F_GC_0.38_avg_30.1945_std_6.63121_Tm_28.95 | AACTCTAGTGAGT |
| HIV_all_2016LANL_WestAfricanGroupM_3571seqs_4376_F_GC_0.46_avg_30.1945_std_6.63121_Tm_32.10 | CTCTCAAAACAGG |
| HIV_all_2016LANL_WestAfricanGroupM_3571seqs_4364_F_GC_0.62_avg_30.1945_std_6.63121_Tm_38.41 | TGGAGACACCTGG |
| HIV_all_2016LANL_WestAfricanGroupM_3571seqs_4352_F_GC_0.23_avg_30.1945_std_6.63121_Tm_22.64 | AATAAACTGGTTA |
| HIV_all_2016LANL_WestAfricanGroupM_3571seqs_4340_F_GC_0.62_avg_30.1945_std_6.63121_Tm_38.41 | GTCAGCCTAGGAC |
| HIV_all_2016LANL_WestAfricanGroupM_3571seqs_4328_F_GC_0.31_avg_30.1945_std_6.63121_Tm_25.79 | CATAGTAATTGGA |
| HIV_all_2016LANL_WestAfricanGroupM_3571seqs_4316_F_GC_0.38_avg_30.1945_std_6.63121_Tm_28.95 | GATAGACAGGTTA |
| HIV_all_2016LANL_WestAfricanGroupM_3571seqs_4304_F_GC_0.46_avg_30.1945_std_6.63121_Tm_32.10 | CAGCTATAGGTTG |
| HIV_all_2016LANL_WestAfricanGroupM_3571seqs_4292_F_GC_0.38_avg_30.1945_std_6.63121_Tm_28.95 | ATCTGTATGTAGG |
| HIV_all_2016LANL_WestAfricanGroupM_3571seqs_4281_F_GC_0.62_avg_30.1945_std_6.63121_Tm_38.41 | GAGAGCTTCGGGT |
| HIV_all_2016LANL_WestAfricanGroupM_3571seqs_4270_F_GC_0.46_avg_30.1945_std_6.63121_Tm_32.10 | CGAAAGTAGAACC |
| HIV_all_2016LANL_WestAfricanGroupM_3571seqs_4259_F_GC_0.62_avg_30.1945_std_6.63121_Tm_38.41 | ATGGGTGCGAGAG |
| HIV_all_2016LANL_WestAfricanGroupM_3571seqs_4248_F_GC_0.46_avg_30.1945_std_6.63121_Tm_32.10 | TAGAGCAGATGCA |
| HIV_all_2016LANL_WestAfricanGroupM_3571seqs_4237_F_GC_0.46_avg_30.1945_std_6.63121_Tm_32.10 | CCTCAAATCACTC |
| HIV_all_2016LANL_WestAfricanGroupM_3571seqs_4226_F_GC_0.23_avg_30.1945_std_6.63121_Tm_22.64 | GAAATACTTGAAA |
| HIV_all_2016LANL_WestAfricanGroupM_3571seqs_4215_F_GC_0.62_avg_30.1945_std_6.63121_Tm_38.41 | GAGTAGGAGGACC |
| HIV_all_2016LANL_WestAfricanGroupM_3571seqs_4204_F_GC_0.38_avg_30.1945_std_6.63121_Tm_28.95 | ATCAGCAAATCCT |
| HIV_all_2016LANL_WestAfricanGroupM_3571seqs_4193_F_GC_0.31_avg_30.1945_std_6.63121_Tm_25.79 | AATATAGTGACAG |
| HIV_all_2016LANL_WestAfricanGroupM_3571seqs_4182_F_GC_0.38_avg_30.1945_std_6.63121_Tm_28.95 | CAGACAATACCAA |
| HIV_all_2016LANL_WestAfricanGroupM_3571seqs_4171_F_GC_0.38_avg_30.1945_std_6.63121_Tm_28.95 | CTAAAACTGCTTG |
| HIV_all_2016LANL_WestAfricanGroupM_3571seqs_4160_F_GC_0.31_avg_30.1945_std_6.63121_Tm_25.79 | AGTAAGGATGTAT |
| HIV_all_2016LANL_WestAfricanGroupM_3571seqs_4149_F_GC_0.46_avg_30.1945_std_6.63121_Tm_32.10 | AGGAGGAAACATG |
| HIV_all_2016LANL_WestAfricanGroupM_3571seqs_4138_F_GC_0.38_avg_30.1945_std_6.63121_Tm_28.95 | CCTATAGTACAGA |
| HIV_all_2016LANL_WestAfricanGroupM_3571seqs_4127_F_GC_0.62_avg_30.1945_std_6.63121_Tm_38.41 | TGTGCTCCTGCTG |
| HIV_all_2016LANL_WestAfricanGroupM_3571seqs_4116_F_GC_0.23_avg_30.1945_std_6.63121_Tm_22.64 | TTCTACTGTAATA |
| HIV_all_2016LANL_WestAfricanGroupM_3571seqs_4105_F_GC_0.23_avg_30.1945_std_6.63121_Tm_22.64 | TGAAATGTAATGA |
| HIV_all_2016LANL_WestAfricanGroupM_3571seqs_4094_F_GC_0.46_avg_30.1945_std_6.63121_Tm_32.10 | AAGCTGAACACCT |
| HIV_all_2016LANL_WestAfricanGroupM_3571seqs_4083_F_GC_0.15_avg_30.1945_std_6.63121_Tm_19.48 | TATAGATTGATAA |
| HIV_all_2016LANL_WestAfricanGroupM_3571seqs_4072_F_GC_0.31_avg_30.1945_std_6.63121_Tm_25.79 | ACATTTAGACCAA |
| HIV_all_2016LANL_WestAfricanGroupM_3571seqs_4061_F_GC_0.23_avg_30.1945_std_6.63121_Tm_22.64 | TCTATAACACTTA |
| HIV_all_2016LANL_WestAfricanGroupM_3571seqs_41615_R_GC_0.62_avg_30.8974_std_6.02042_Tm_38.41 | AGCAGTGGGTTCC |
| HIV_all_2016LANL_WestAfricanGroupM_3571seqs_40483_R_GC_0.46_avg_30.8974_std_6.02042_Tm_32.10 | TTCTTCCTGCCAT |
| HIV_all_2016LANL_WestAfricanGroupM_3571seqs_39613_R_GC_0.46_avg_30.8974_std_6.02042_Tm_32.10 | CATTGACAGTCCA |
| HIV_all_2016LANL_WestAfricanGroupM_3571seqs_38790_R_GC_0.62_avg_30.8974_std_6.02042_Tm_38.41 | CCCTGCCTAACTC |
| HIV_all_2016LANL_WestAfricanGroupM_3571seqs_38073_R_GC_0.54_avg_30.8974_std_6.02042_Tm_35.25 | TCAGGAATCCAGG |
| HIV_all_2016LANL_WestAfricanGroupM_3571seqs_37363_R_GC_0.31_avg_30.8974_std_6.02042_Tm_25.79 | GCTATTATGTCTA |
| HIV_all_2016LANL_WestAfricanGroupM_3571seqs_35949_R_GC_0.54_avg_30.8974_std_6.02042_Tm_35.25 | TCTGTGGGTACAC |
| HIV_all_2016LANL_WestAfricanGroupM_3571seqs_35252_R_GC_0.38_avg_30.8974_std_6.02042_Tm_28.95 | TCTGATAATGCTG |
| HIV_all_2016LANL_WestAfricanGroupM_3571seqs_34557_R_GC_0.54_avg_30.8974_std_6.02042_Tm_35.25 | TTCCAGGGCTCTA |
| HIV_all_2016LANL_WestAfricanGroupM_3571seqs_33886_R_GC_0.62_avg_30.8974_std_6.02042_Tm_38.41 | TTGCCTGGAGCTG |
| HIV_all_2016LANL_WestAfricanGroupM_3571seqs_33233_R_GC_0.38_avg_30.8974_std_6.02042_Tm_28.95 | ACCCCAATATGTT |
| HIV_all_2016LANL_WestAfricanGroupM_3571seqs_32589_R_GC_0.54_avg_30.8974_std_6.02042_Tm_35.25 | CACTGGCTACATG |
| HIV_all_2016LANL_WestAfricanGroupM_3571seqs_31948_R_GC_0.23_avg_30.8974_std_6.02042_Tm_22.64 | AAAAATATGCATC |
| HIV_all_2016LANL_WestAfricanGroupM_3571seqs_31318_R_GC_0.38_avg_30.8974_std_6.02042_Tm_28.95 | TTTGATGGGTCAT |
| HIV_all_2016LANL_WestAfricanGroupM_3571seqs_30700_R_GC_0.54_avg_30.8974_std_6.02042_Tm_35.25 | GGTGGTAGCTGAA |
| HIV_all_2016LANL_WestAfricanGroupM_3571seqs_30103_R_GC_0.38_avg_30.8974_std_6.02042_Tm_28.95 | ACTGTACCTATAG |
| HIV_all_2016LANL_WestAfricanGroupM_3571seqs_29517_R_GC_0.46_avg_30.8974_std_6.02042_Tm_32.10 | TATGTCCTGCTTG |
| HIV_all_2016LANL_WestAfricanGroupM_3571seqs_28969_R_GC_0.38_avg_30.8974_std_6.02042_Tm_28.95 | TTTTGACCACTTG |
| HIV_all_2016LANL_WestAfricanGroupM_3571seqs_28425_R_GC_0.38_avg_30.8974_std_6.02042_Tm_28.95 | CTTTTCTTCTTGG |
| HIV_all_2016LANL_WestAfricanGroupM_3571seqs_27882_R_GC_0.46_avg_30.8974_std_6.02042_Tm_32.10 | TGTCAGCAGTCTT |
| HIV_all_2016LANL_WestAfricanGroupM_3571seqs_27356_R_GC_0.38_avg_30.8974_std_6.02042_Tm_28.95 | TACAATCTGGGTT |
| HIV_all_2016LANL_WestAfricanGroupM_3571seqs_26851_R_GC_0.38_avg_30.8974_std_6.02042_Tm_28.95 | TGTCAAACTTCCA |
| HIV_all_2016LANL_WestAfricanGroupM_3571seqs_26373_R_GC_0.46_avg_30.8974_std_6.02042_Tm_32.10 | ATCCAGGTCATGT |
| HIV_all_2016LANL_WestAfricanGroupM_3571seqs_25912_R_GC_0.54_avg_30.8974_std_6.02042_Tm_35.25 | ACTGGGATAGGTG |
| HIV_all_2016LANL_WestAfricanGroupM_3571seqs_25455_R_GC_0.46_avg_30.8974_std_6.02042_Tm_32.10 | GTCTAACAGCTTC |
| HIV_all_2016LANL_WestAfricanGroupM_3571seqs_25002_R_GC_0.69_avg_30.8974_std_6.02042_Tm_41.56 | TCCCCTCCTGAGG |
| HIV_all_2016LANL_WestAfricanGroupM_3571seqs_24552_R_GC_0.46_avg_30.8974_std_6.02042_Tm_32.10 | CTTCTTTGCCACA |
| HIV_all_2016LANL_WestAfricanGroupM_3571seqs_24104_R_GC_0.31_avg_30.8974_std_6.02042_Tm_25.79 | TATCTATTCCATC |
| HIV_all_2016LANL_WestAfricanGroupM_3571seqs_23657_R_GC_0.38_avg_30.8974_std_6.02042_Tm_28.95 | CTGCCATTTAACA |
| HIV_all_2016LANL_WestAfricanGroupM_3571seqs_23242_R_GC_0.23_avg_30.8974_std_6.02042_Tm_22.64 | ATGTATTGATAGA |
| HIV_all_2016LANL_WestAfricanGroupM_3571seqs_22830_R_GC_0.69_avg_30.8974_std_6.02042_Tm_41.56 | CCAGAGAGCTCCC |
| HIV_all_2016LANL_WestAfricanGroupM_3571seqs_22421_R_GC_0.54_avg_30.8974_std_6.02042_Tm_35.25 | AAATTCCCTGGCC |
| HIV_all_2016LANL_WestAfricanGroupM_3571seqs_22017_R_GC_0.31_avg_30.8974_std_6.02042_Tm_25.79 | ACAACTATTGCTA |
| HIV_all_2016LANL_WestAfricanGroupM_3571seqs_21637_R_GC_0.46_avg_30.8974_std_6.02042_Tm_32.10 | ATCCTGAATCCTG |
| HIV_all_2016LANL_WestAfricanGroupM_3571seqs_21265_R_GC_0.38_avg_30.8974_std_6.02042_Tm_28.95 | CCATCTCTTGTTA |
| HIV_all_2016LANL_WestAfricanGroupM_3571seqs_20909_R_GC_0.54_avg_30.8974_std_6.02042_Tm_35.25 | GTCCTGGTCCTAT |
| HIV_all_2016LANL_WestAfricanGroupM_3571seqs_20555_R_GC_0.54_avg_30.8974_std_6.02042_Tm_35.25 | CCACAGATCAAGG |
| HIV_all_2016LANL_WestAfricanGroupM_3571seqs_20227_R_GC_0.54_avg_30.8974_std_6.02042_Tm_35.25 | TCTGAGGGATCTC |
| HIV_all_2016LANL_WestAfricanGroupM_3571seqs_19900_R_GC_0.46_avg_30.8974_std_6.02042_Tm_32.10 | GCTGCTTATATGC |
| HIV_all_2016LANL_WestAfricanGroupM_3571seqs_19614_R_GC_0.38_avg_30.8974_std_6.02042_Tm_28.95 | ATAACCCTATCTG |
| HIV_all_2016LANL_WestAfricanGroupM_3571seqs_19350_R_GC_0.31_avg_30.8974_std_6.02042_Tm_25.79 | ATAATCCTATCTG |
| HIV_all_2016LANL_WestAfricanGroupM_3571seqs_19107_R_GC_0.31_avg_30.8974_std_6.02042_Tm_25.79 | GTACTATATCAAG |
| HIV_all_2016LANL_WestAfricanGroupM_3571seqs_18870_R_GC_0.62_avg_30.8974_std_6.02042_Tm_38.41 | GCGGAAAGTCCCT |
| HIV_all_2016LANL_WestAfricanGroupM_3571seqs_18636_R_GC_0.46_avg_30.8974_std_6.02042_Tm_32.10 | TGCCACAGTTGAA |
| HIV_all_2016LANL_WestAfricanGroupM_3571seqs_18404_R_GC_0.46_avg_30.8974_std_6.02042_Tm_32.10 | CTAAAGGGCTCTA |
| HIV_all_2016LANL_WestAfricanGroupM_3571seqs_18178_R_GC_0.54_avg_30.8974_std_6.02042_Tm_35.25 | ATCCCAAGGAGCA |
| HIV_all_2016LANL_WestAfricanGroupM_3571seqs_17959_R_GC_0.54_avg_30.8974_std_6.02042_Tm_35.25 | GTCTGACAGCTTC |
| HIV_all_2016LANL_WestAfricanGroupM_3571seqs_17742_R_GC_0.23_avg_30.8974_std_6.02042_Tm_22.64 | CCATCTAAAAATA |
| HIV_all_2016LANL_WestAfricanGroupM_3571seqs_17528_R_GC_0.46_avg_30.8974_std_6.02042_Tm_32.10 | GTCATCCATGCTA |
| HIV_all_2016LANL_WestAfricanGroupM_3571seqs_17323_R_GC_0.31_avg_30.8974_std_6.02042_Tm_25.79 | ATTTTTCCTTCCT |
| HIV_all_2016LANL_WestAfricanGroupM_3571seqs_17123_R_GC_0.54_avg_30.8974_std_6.02042_Tm_35.25 | AGCCTTGTGTGTG |
| HIV_all_2016LANL_WestAfricanGroupM_3571seqs_16925_R_GC_0.31_avg_30.8974_std_6.02042_Tm_25.79 | CTACCATTTAACA |
| HIV_all_2016LANL_WestAfricanGroupM_3571seqs_16729_R_GC_0.46_avg_30.8974_std_6.02042_Tm_32.10 | TGGTTGTAGCTGT |
| HIV_all_2016LANL_WestAfricanGroupM_3571seqs_16533_R_GC_0.31_avg_30.8974_std_6.02042_Tm_25.79 | GAATTGCTTGTAA |
| HIV_all_2016LANL_WestAfricanGroupM_3571seqs_16344_R_GC_0.23_avg_30.8974_std_6.02042_Tm_22.64 | CTTTTTCTTGTAT |
| HIV_all_2016LANL_WestAfricanGroupM_3571seqs_16160_R_GC_0.62_avg_30.8974_std_6.02042_Tm_38.41 | GAAGAGGCACAGG |
| HIV_all_2016LANL_WestAfricanGroupM_3571seqs_15983_R_GC_0.31_avg_30.8974_std_6.02042_Tm_25.79 | GTACTACCTTTAT |
| HIV_all_2016LANL_WestAfricanGroupM_3571seqs_15809_R_GC_0.54_avg_30.8974_std_6.02042_Tm_35.25 | CTGCTACCAGGAT |
| HIV_all_2016LANL_WestAfricanGroupM_3571seqs_15641_R_GC_0.46_avg_30.8974_std_6.02042_Tm_32.10 | CTGGATTCGCATT |
| HIV_all_2016LANL_WestAfricanGroupM_3571seqs_15477_R_GC_0.69_avg_30.8974_std_6.02042_Tm_41.56 | CTGGCCTCCCCTT |
| HIV_all_2016LANL_WestAfricanGroupM_3571seqs_15314_R_GC_0.15_avg_30.8974_std_6.02042_Tm_19.48 | TAATTGCTAATTT |
| HIV_all_2016LANL_WestAfricanGroupM_3571seqs_15153_R_GC_0.15_avg_30.8974_std_6.02042_Tm_19.48 | TCAAGTTTATAAA |
| HIV_all_2016LANL_WestAfricanGroupM_3571seqs_14995_R_GC_0.38_avg_30.8974_std_6.02042_Tm_28.95 | TCCAAAAGCCTAA |
| HIV_all_2016LANL_WestAfricanGroupM_3571seqs_14837_R_GC_0.62_avg_30.8974_std_6.02042_Tm_38.41 | TAGCCACTCCCCA |
| HIV_all_2016LANL_WestAfricanGroupM_3571seqs_14680_R_GC_0.46_avg_30.8974_std_6.02042_Tm_32.10 | TTAATGCCCCAGA |
| HIV_all_2016LANL_WestAfricanGroupM_3571seqs_14523_R_GC_0.46_avg_30.8974_std_6.02042_Tm_32.10 | GATCCTACCTTGT |
| HIV_all_2016LANL_WestAfricanGroupM_3571seqs_14368_R_GC_0.31_avg_30.8974_std_6.02042_Tm_25.79 | CCACAAATTTCTA |
| HIV_all_2016LANL_WestAfricanGroupM_3571seqs_14224_R_GC_0.46_avg_30.8974_std_6.02042_Tm_32.10 | CTATAGTCCACAC |
| HIV_all_2016LANL_WestAfricanGroupM_3571seqs_14083_R_GC_0.31_avg_30.8974_std_6.02042_Tm_25.79 | TGTATTTCTGCTA |
| HIV_all_2016LANL_WestAfricanGroupM_3571seqs_13948_R_GC_0.54_avg_30.8974_std_6.02042_Tm_35.25 | AGAGACCCAGTAC |
| HIV_all_2016LANL_WestAfricanGroupM_3571seqs_13813_R_GC_0.54_avg_30.8974_std_6.02042_Tm_35.25 | CTATGGTCCACAC |
| HIV_all_2016LANL_WestAfricanGroupM_3571seqs_13680_R_GC_0.54_avg_30.8974_std_6.02042_Tm_35.25 | AGTACTCCGGATG |
| HIV_all_2016LANL_WestAfricanGroupM_3571seqs_13549_R_GC_0.31_avg_30.8974_std_6.02042_Tm_25.79 | CCTGTAATATTTG |
| HIV_all_2016LANL_WestAfricanGroupM_3571seqs_13419_R_GC_0.62_avg_30.8974_std_6.02042_Tm_38.41 | TCTTGTGGGGTGG |
| HIV_all_2016LANL_WestAfricanGroupM_3571seqs_13289_R_GC_0.46_avg_30.8974_std_6.02042_Tm_32.10 | ATCACCAACCCAA |
| HIV_all_2016LANL_WestAfricanGroupM_3571seqs_13159_R_GC_0.62_avg_30.8974_std_6.02042_Tm_38.41 | TAGCCTCCGCTAG |
| HIV_all_2016LANL_WestAfricanGroupM_3571seqs_13036_R_GC_0.62_avg_30.8974_std_6.02042_Tm_38.41 | TCTCCTCCTGAGG |
| HIV_all_2016LANL_WestAfricanGroupM_3571seqs_12913_R_GC_0.31_avg_30.8974_std_6.02042_Tm_25.79 | TTTTGACCATTTG |
| HIV_all_2016LANL_WestAfricanGroupM_3571seqs_12798_R_GC_0.38_avg_30.8974_std_6.02042_Tm_28.95 | TCAAGGATCTCTT |
| HIV_all_2016LANL_WestAfricanGroupM_3571seqs_12684_R_GC_0.38_avg_30.8974_std_6.02042_Tm_28.95 | TGTATGTCTGTTG |
| HIV_all_2016LANL_WestAfricanGroupM_3571seqs_12573_R_GC_0.31_avg_30.8974_std_6.02042_Tm_25.79 | CACAATTGAAACA |
| HIV_all_2016LANL_WestAfricanGroupM_3571seqs_12462_R_GC_0.62_avg_30.8974_std_6.02042_Tm_38.41 | GGCCTTCCCTTGT |
| HIV_all_2016LANL_WestAfricanGroupM_3571seqs_12354_R_GC_0.38_avg_30.8974_std_6.02042_Tm_28.95 | GATTCTACTACTC |
| HIV_all_2016LANL_WestAfricanGroupM_3571seqs_12250_R_GC_0.54_avg_30.8974_std_6.02042_Tm_35.25 | GTGGCTTGCCAAT |
| HIV_all_2016LANL_WestAfricanGroupM_3571seqs_12146_R_GC_0.46_avg_30.8974_std_6.02042_Tm_32.10 | CTAGACTGCCATT |
| HIV_all_2016LANL_WestAfricanGroupM_3571seqs_12043_R_GC_0.46_avg_30.8974_std_6.02042_Tm_32.10 | GTCATCCATCCTA |
| HIV_all_2016LANL_WestAfricanGroupM_3571seqs_11941_R_GC_0.46_avg_30.8974_std_6.02042_Tm_32.10 | TAGGATCTACTGG |
| HIV_all_2016LANL_WestAfricanGroupM_3571seqs_11738_R_GC_0.38_avg_30.8974_std_6.02042_Tm_28.95 | TATCTATCCCATC |
| HIV_all_2016LANL_WestAfricanGroupM_3571seqs_11637_R_GC_0.46_avg_30.8974_std_6.02042_Tm_32.10 | CTGGTGTGTAGTT |
| HIV_all_2016LANL_WestAfricanGroupM_3571seqs_11536_R_GC_0.69_avg_30.8974_std_6.02042_Tm_41.56 | CAGCTGCTGCCTG |
| HIV_all_2016LANL_WestAfricanGroupM_3571seqs_11436_R_GC_0.38_avg_30.8974_std_6.02042_Tm_28.95 | GGCAAGCTTTATT |
| HIV_all_2016LANL_WestAfricanGroupM_3571seqs_11338_R_GC_0.31_avg_30.8974_std_6.02042_Tm_25.79 | TGTAATTCAGTCT |
| HIV_all_2016LANL_WestAfricanGroupM_3571seqs_11240_R_GC_0.54_avg_30.8974_std_6.02042_Tm_35.25 | GCATCACCCACAT |
| HIV_all_2016LANL_WestAfricanGroupM_3571seqs_11145_R_GC_0.62_avg_30.8974_std_6.02042_Tm_38.41 | CCCCTCAGCTACT |
| HIV_all_2016LANL_WestAfricanGroupM_3571seqs_11054_R_GC_0.54_avg_30.8974_std_6.02042_Tm_35.25 | TCTCTCTGCACCA |
| HIV_all_2016LANL_WestAfricanGroupM_3571seqs_10965_R_GC_0.15_avg_30.8974_std_6.02042_Tm_19.48 | AAAAACTATTCTT |
| HIV_all_2016LANL_WestAfricanGroupM_3571seqs_10877_R_GC_0.38_avg_30.8974_std_6.02042_Tm_28.95 | TCTGGATTTGCAT |
| HIV_all_2016LANL_WestAfricanGroupM_3571seqs_10791_R_GC_0.38_avg_30.8974_std_6.02042_Tm_28.95 | CCTGTGATATTTG |
| HIV_all_2016LANL_WestAfricanGroupM_3571seqs_10705_R_GC_0.69_avg_30.8974_std_6.02042_Tm_41.56 | GTGGGTTGGGGTC |
| HIV_all_2016LANL_WestAfricanGroupM_3571seqs_10619_R_GC_0.54_avg_30.8974_std_6.02042_Tm_35.25 | CAGCCTTCTGATG |
| HIV_all_2016LANL_WestAfricanGroupM_3571seqs_10534_R_GC_0.54_avg_30.8974_std_6.02042_Tm_35.25 | CCCTGGTCCTATA |
| HIV_all_2016LANL_WestAfricanGroupM_3571seqs_10449_R_GC_0.38_avg_30.8974_std_6.02042_Tm_28.95 | TCTGCTGTTTCTA |
| HIV_all_2016LANL_WestAfricanGroupM_3571seqs_10366_R_GC_0.46_avg_30.8974_std_6.02042_Tm_32.10 | AGCCTTGTGTGTT |
| HIV_all_2016LANL_WestAfricanGroupM_3571seqs_10285_R_GC_0.54_avg_30.8974_std_6.02042_Tm_35.25 | CCACCACTCTTCT |
| HIV_all_2016LANL_WestAfricanGroupM_3571seqs_10204_R_GC_0.38_avg_30.8974_std_6.02042_Tm_28.95 | TGGTACTACATCA |
| HIV_all_2016LANL_WestAfricanGroupM_3571seqs_10124_R_GC_0.31_avg_30.8974_std_6.02042_Tm_25.79 | GTTTTCTAAAAGG |
| HIV_all_2016LANL_WestAfricanGroupM_3571seqs_10045_R_GC_0.31_avg_30.8974_std_6.02042_Tm_25.79 | CATAATCCCTAAT |
| HIV_all_2016LANL_WestAfricanGroupM_3571seqs_9967_R_GC_0.31_avg_30.8974_std_6.02042_Tm_25.79 | GTGCAATTTAAAG |
| HIV_all_2016LANL_WestAfricanGroupM_3571seqs_9893_R_GC_0.46_avg_30.8974_std_6.02042_Tm_32.10 | CTGTCAAATCTCC |
| HIV_all_2016LANL_WestAfricanGroupM_3571seqs_9823_R_GC_0.62_avg_30.8974_std_6.02042_Tm_38.41 | TCTAGGTCCCCTC |
| HIV_all_2016LANL_WestAfricanGroupM_3571seqs_9754_R_GC_0.46_avg_30.8974_std_6.02042_Tm_32.10 | TCTGTTCCTTCAG |
| HIV_all_2016LANL_WestAfricanGroupM_3571seqs_9686_R_GC_0.54_avg_30.8974_std_6.02042_Tm_35.25 | CTAGTGGGATGTG |
| HIV_all_2016LANL_WestAfricanGroupM_3571seqs_9618_R_GC_0.46_avg_30.8974_std_6.02042_Tm_32.10 | GCCTAGGAAAATG |
| HIV_all_2016LANL_WestAfricanGroupM_3571seqs_9550_R_GC_0.46_avg_30.8974_std_6.02042_Tm_32.10 | TCAGCAGTCTTTG |
| HIV_all_2016LANL_WestAfricanGroupM_3571seqs_9483_R_GC_0.38_avg_30.8974_std_6.02042_Tm_28.95 | GTAACTCAGTCTT |
| HIV_all_2016LANL_WestAfricanGroupM_3571seqs_9417_R_GC_0.38_avg_30.8974_std_6.02042_Tm_28.95 | GATCCTACCTTAT |
| HIV_all_2016LANL_WestAfricanGroupM_3571seqs_9351_R_GC_0.46_avg_30.8974_std_6.02042_Tm_32.10 | ATTCCAAGGAGCA |
| HIV_all_2016LANL_WestAfricanGroupM_3571seqs_9286_R_GC_0.62_avg_30.8974_std_6.02042_Tm_38.41 | CCCACTGCATCCA |
| HIV_all_2016LANL_WestAfricanGroupM_3571seqs_9222_R_GC_0.23_avg_30.8974_std_6.02042_Tm_22.64 | GTACAATTTAAAG |
| HIV_all_2016LANL_WestAfricanGroupM_3571seqs_9158_R_GC_0.62_avg_30.8974_std_6.02042_Tm_38.41 | CTGGAAAGTCCCC |
| HIV_all_2016LANL_WestAfricanGroupM_3571seqs_9094_R_GC_0.46_avg_30.8974_std_6.02042_Tm_32.10 | TGGAAGGCCAAAT |
| HIV_all_2016LANL_WestAfricanGroupM_3571seqs_9032_R_GC_0.15_avg_30.8974_std_6.02042_Tm_19.48 | CAATATGTTTTTA |
| HIV_all_2016LANL_WestAfricanGroupM_3571seqs_8972_R_GC_0.46_avg_30.8974_std_6.02042_Tm_32.10 | CCTGAATCTTGCA |
| HIV_all_2016LANL_WestAfricanGroupM_3571seqs_8914_R_GC_0.23_avg_30.8974_std_6.02042_Tm_22.64 | AAAAGTGCATATT |
| HIV_all_2016LANL_WestAfricanGroupM_3571seqs_8856_R_GC_0.62_avg_30.8974_std_6.02042_Tm_38.41 | CATCCCCCACATC |
| HIV_all_2016LANL_WestAfricanGroupM_3571seqs_8799_R_GC_0.31_avg_30.8974_std_6.02042_Tm_25.79 | TTAAAAAGTGGCT |
| HIV_all_2016LANL_WestAfricanGroupM_3571seqs_8742_R_GC_0.69_avg_30.8974_std_6.02042_Tm_41.56 | TCTGGGTCCCCTC |
| HIV_all_2016LANL_WestAfricanGroupM_3571seqs_8687_R_GC_0.15_avg_30.8974_std_6.02042_Tm_19.48 | ATGCTTTTAAAAT |
| HIV_all_2016LANL_WestAfricanGroupM_3571seqs_8633_R_GC_0.15_avg_30.8974_std_6.02042_Tm_19.48 | CAATTTTTGAAAT |
| HIV_all_2016LANL_WestAfricanGroupM_3571seqs_8579_R_GC_0.38_avg_30.8974_std_6.02042_Tm_28.95 | TTCTTTCCCTTAC |
| HIV_all_2016LANL_WestAfricanGroupM_3571seqs_8526_R_GC_0.46_avg_30.8974_std_6.02042_Tm_32.10 | GGAGTGTGATAGT |
| HIV_all_2016LANL_WestAfricanGroupM_3571seqs_8474_R_GC_0.46_avg_30.8974_std_6.02042_Tm_32.10 | GTGATTGCTCCAT |
| HIV_all_2016LANL_WestAfricanGroupM_3571seqs_8422_R_GC_0.23_avg_30.8974_std_6.02042_Tm_22.64 | TTCAGATCTAATT |
| HIV_all_2016LANL_WestAfricanGroupM_3571seqs_8371_R_GC_0.54_avg_30.8974_std_6.02042_Tm_35.25 | TTTCTCCCACTGG |
| HIV_all_2016LANL_WestAfricanGroupM_3571seqs_8321_R_GC_0.46_avg_30.8974_std_6.02042_Tm_32.10 | TTCTGTGTCAGCA |
| HIV_all_2016LANL_WestAfricanGroupM_3571seqs_8273_R_GC_0.38_avg_30.8974_std_6.02042_Tm_28.95 | CCTTGTTTCTGTA |
| HIV_all_2016LANL_WestAfricanGroupM_3571seqs_8224_R_GC_0.38_avg_30.8974_std_6.02042_Tm_28.95 | CCACAGATTTCTA |
| HIV_all_2016LANL_WestAfricanGroupM_3571seqs_8176_R_GC_0.38_avg_30.8974_std_6.02042_Tm_28.95 | GGAGTGTGATATT |
| HIV_all_2016LANL_WestAfricanGroupM_3571seqs_8128_R_GC_0.38_avg_30.8974_std_6.02042_Tm_28.95 | GGAAGTATGATAG |
| HIV_all_2016LANL_WestAfricanGroupM_3571seqs_8080_R_GC_0.31_avg_30.8974_std_6.02042_Tm_25.79 | CTATTTCTGGATT |
| HIV_all_2016LANL_WestAfricanGroupM_3571seqs_8032_R_GC_0.46_avg_30.8974_std_6.02042_Tm_32.10 | CTCTATCTGTCCA |
| HIV_all_2016LANL_WestAfricanGroupM_3571seqs_7985_R_GC_0.31_avg_30.8974_std_6.02042_Tm_25.79 | CCTTGTGTATTAT |
| HIV_all_2016LANL_WestAfricanGroupM_3571seqs_7938_R_GC_0.31_avg_30.8974_std_6.02042_Tm_25.79 | TCTTTATCCCATT |
| HIV_all_2016LANL_WestAfricanGroupM_3571seqs_7892_R_GC_0.38_avg_30.8974_std_6.02042_Tm_28.95 | GTGCCTTTTTCTT |
| HIV_all_2016LANL_WestAfricanGroupM_3571seqs_7846_R_GC_0.69_avg_30.8974_std_6.02042_Tm_41.56 | CGGCTCCTGCTTC |
| HIV_all_2016LANL_WestAfricanGroupM_3571seqs_7802_R_GC_0.23_avg_30.8974_std_6.02042_Tm_22.64 | CAAGTCTATAAAA |
| HIV_all_2016LANL_WestAfricanGroupM_3571seqs_7760_R_GC_0.31_avg_30.8974_std_6.02042_Tm_25.79 | AAAAATATGCGTC |
| HIV_all_2016LANL_WestAfricanGroupM_3571seqs_7718_R_GC_0.23_avg_30.8974_std_6.02042_Tm_22.64 | ACAATTATTGCTA |
| HIV_all_2016LANL_WestAfricanGroupM_3571seqs_7676_R_GC_0.46_avg_30.8974_std_6.02042_Tm_32.10 | GGAGTGTGATTGT |
| HIV_all_2016LANL_WestAfricanGroupM_3571seqs_7634_R_GC_0.62_avg_30.8974_std_6.02042_Tm_38.41 | AGGCGGCTGTCAA |
| HIV_all_2016LANL_WestAfricanGroupM_3571seqs_7550_R_GC_0.38_avg_30.8974_std_6.02042_Tm_28.95 | ACTTTCCTGATTC |
| HIV_all_2016LANL_WestAfricanGroupM_3571seqs_7508_R_GC_0.62_avg_30.8974_std_6.02042_Tm_38.41 | TCCCACTCCATCC |
| HIV_all_2016LANL_WestAfricanGroupM_3571seqs_7466_R_GC_0.62_avg_30.8974_std_6.02042_Tm_38.41 | CCCCAGACTGTGA |
| HIV_all_2016LANL_WestAfricanGroupM_3571seqs_7425_R_GC_0.38_avg_30.8974_std_6.02042_Tm_28.95 | AAAGTGACACAGA |
| HIV_all_2016LANL_WestAfricanGroupM_3571seqs_7385_R_GC_0.46_avg_30.8974_std_6.02042_Tm_32.10 | CTATCTGTTCCCT |
| HIV_all_2016LANL_WestAfricanGroupM_3571seqs_7346_R_GC_0.54_avg_30.8974_std_6.02042_Tm_35.25 | AGGCTGCTGTCAA |
| HIV_all_2016LANL_WestAfricanGroupM_3571seqs_7307_R_GC_0.38_avg_30.8974_std_6.02042_Tm_28.95 | CCTGTAATGTTTG |
| HIV_all_2016LANL_WestAfricanGroupM_3571seqs_7268_R_GC_0.54_avg_30.8974_std_6.02042_Tm_35.25 | CCATCTCCACAAG |
| HIV_all_2016LANL_WestAfricanGroupM_3571seqs_7230_R_GC_0.31_avg_30.8974_std_6.02042_Tm_25.79 | TTGGTACTACTTT |
| HIV_all_2016LANL_WestAfricanGroupM_3571seqs_7193_R_GC_0.23_avg_30.8974_std_6.02042_Tm_22.64 | TGTTGCATAAAAT |
| HIV_all_2016LANL_WestAfricanGroupM_3571seqs_7156_R_GC_0.23_avg_30.8974_std_6.02042_Tm_22.64 | TTGTTTTGTTCTT |
| HIV_all_2016LANL_WestAfricanGroupM_3571seqs_7119_R_GC_0.38_avg_30.8974_std_6.02042_Tm_28.95 | TTGTTGACATCCT |
| HIV_all_2016LANL_WestAfricanGroupM_3571seqs_7082_R_GC_0.23_avg_30.8974_std_6.02042_Tm_22.64 | TCAAGCTTATAAA |
| HIV_all_2016LANL_WestAfricanGroupM_3571seqs_7046_R_GC_0.38_avg_30.8974_std_6.02042_Tm_28.95 | GGAGAGTTATAGT |
| HIV_all_2016LANL_WestAfricanGroupM_3571seqs_7010_R_GC_0.54_avg_30.8974_std_6.02042_Tm_35.25 | CCCCATCTCCATA |
| HIV_all_2016LANL_WestAfricanGroupM_3571seqs_6974_R_GC_0.15_avg_30.8974_std_6.02042_Tm_19.48 | AGCAAAAATTATT |
| HIV_all_2016LANL_WestAfricanGroupM_3571seqs_6939_R_GC_0.54_avg_30.8974_std_6.02042_Tm_35.25 | CCAGGCACAATCA |
| HIV_all_2016LANL_WestAfricanGroupM_3571seqs_6904_R_GC_0.46_avg_30.8974_std_6.02042_Tm_32.10 | CTAGGAAAGTGTC |
| HIV_all_2016LANL_WestAfricanGroupM_3571seqs_6869_R_GC_0.54_avg_30.8974_std_6.02042_Tm_35.25 | TCCGGATGTAGCT |
| HIV_all_2016LANL_WestAfricanGroupM_3571seqs_6835_R_GC_0.54_avg_30.8974_std_6.02042_Tm_35.25 | CCTGCCAATACTC |
| HIV_all_2016LANL_WestAfricanGroupM_3571seqs_6801_R_GC_0.15_avg_30.8974_std_6.02042_Tm_19.48 | TTATTCATAGATT |
| HIV_all_2016LANL_WestAfricanGroupM_3571seqs_6767_R_GC_0.54_avg_30.8974_std_6.02042_Tm_35.25 | GGTCTGCTCTGAA |
| HIV_all_2016LANL_WestAfricanGroupM_3571seqs_6733_R_GC_0.15_avg_30.8974_std_6.02042_Tm_19.48 | AAAGATTATTGTT |
| HIV_all_2016LANL_WestAfricanGroupM_3571seqs_6700_R_GC_0.54_avg_30.8974_std_6.02042_Tm_35.25 | CTGGGTTCGCATT |
| HIV_all_2016LANL_WestAfricanGroupM_3571seqs_6634_R_GC_0.46_avg_30.8974_std_6.02042_Tm_32.10 | CCCTGTTTCTGTA |
| HIV_all_2016LANL_WestAfricanGroupM_3571seqs_6601_R_GC_0.38_avg_30.8974_std_6.02042_Tm_28.95 | TGTATGGGAATTG |
| HIV_all_2016LANL_WestAfricanGroupM_3571seqs_6569_R_GC_0.38_avg_30.8974_std_6.02042_Tm_28.95 | GTAAGTCTCTCAA |
| HIV_all_2016LANL_WestAfricanGroupM_3571seqs_6537_R_GC_0.62_avg_30.8974_std_6.02042_Tm_38.41 | GGATGGTTCCAGG |
| HIV_all_2016LANL_WestAfricanGroupM_3571seqs_6505_R_GC_0.62_avg_30.8974_std_6.02042_Tm_38.41 | AGACGGGCACACA |
| HIV_all_2016LANL_WestAfricanGroupM_3571seqs_6474_R_GC_0.31_avg_30.8974_std_6.02042_Tm_25.79 | TCTTGTCTTTTCT |
| HIV_all_2016LANL_WestAfricanGroupM_3571seqs_6443_R_GC_0.54_avg_30.8974_std_6.02042_Tm_35.25 | TATGTCCTGCTGG |
| HIV_all_2016LANL_WestAfricanGroupM_3571seqs_6413_R_GC_0.54_avg_30.8974_std_6.02042_Tm_35.25 | CTTCCTTGCCACA |
| HIV_all_2016LANL_WestAfricanGroupM_3571seqs_6384_R_GC_0.38_avg_30.8974_std_6.02042_Tm_28.95 | ATGAAGTCTCTCA |
| HIV_all_2016LANL_WestAfricanGroupM_3571seqs_6355_R_GC_0.46_avg_30.8974_std_6.02042_Tm_32.10 | CCACTGCTCTTTT |
| HIV_all_2016LANL_WestAfricanGroupM_3571seqs_6326_R_GC_0.46_avg_30.8974_std_6.02042_Tm_32.10 | TATGGCTGCTATG |
| HIV_all_2016LANL_WestAfricanGroupM_3571seqs_6297_R_GC_0.62_avg_30.8974_std_6.02042_Tm_38.41 | CTGCTCCTACTCC |
| HIV_all_2016LANL_WestAfricanGroupM_3571seqs_6269_R_GC_0.31_avg_30.8974_std_6.02042_Tm_25.79 | TGGTACTATATCA |
| HIV_all_2016LANL_WestAfricanGroupM_3571seqs_6241_R_GC_0.46_avg_30.8974_std_6.02042_Tm_32.10 | GGATACAGTTCCT |
| HIV_all_2016LANL_WestAfricanGroupM_3571seqs_6213_R_GC_0.23_avg_30.8974_std_6.02042_Tm_22.64 | CTATTATTCTTTC |
| HIV_all_2016LANL_WestAfricanGroupM_3571seqs_6185_R_GC_0.38_avg_30.8974_std_6.02042_Tm_28.95 | TCTTCTTCTGCTA |
| HIV_all_2016LANL_WestAfricanGroupM_3571seqs_6158_R_GC_0.46_avg_30.8974_std_6.02042_Tm_32.10 | TCCTGCAAAGCTA |
| HIV_all_2016LANL_WestAfricanGroupM_3571seqs_6131_R_GC_0.54_avg_30.8974_std_6.02042_Tm_35.25 | GGCCATCCAACTA |
| HIV_all_2016LANL_WestAfricanGroupM_3571seqs_6104_R_GC_0.38_avg_30.8974_std_6.02042_Tm_28.95 | TGTGTCTTTTGCT |
| HIV_all_2016LANL_WestAfricanGroupM_3571seqs_6077_R_GC_0.31_avg_30.8974_std_6.02042_Tm_25.79 | ATACCTTTTCTTG |
| HIV_all_2016LANL_WestAfricanGroupM_3571seqs_6051_R_GC_0.31_avg_30.8974_std_6.02042_Tm_25.79 | TAGGAAAATGTCT |
| HIV_all_2016LANL_WestAfricanGroupM_3571seqs_6025_R_GC_0.54_avg_30.8974_std_6.02042_Tm_35.25 | TGCCAATCAGGGA |
| HIV_all_2016LANL_WestAfricanGroupM_3571seqs_5999_R_GC_0.46_avg_30.8974_std_6.02042_Tm_32.10 | GGAGTGTGATGTT |
| HIV_all_2016LANL_WestAfricanGroupM_3571seqs_5973_R_GC_0.38_avg_30.8974_std_6.02042_Tm_28.95 | CTGTTAAATCTCC |
| HIV_all_2016LANL_WestAfricanGroupM_3571seqs_5948_R_GC_0.38_avg_30.8974_std_6.02042_Tm_28.95 | AATAGTAGTCCTG |
| HIV_all_2016LANL_WestAfricanGroupM_3571seqs_5923_R_GC_0.46_avg_30.8974_std_6.02042_Tm_32.10 | TCAGCAGTTCTTG |
| HIV_all_2016LANL_WestAfricanGroupM_3571seqs_5898_R_GC_0.54_avg_30.8974_std_6.02042_Tm_35.25 | GGAGTGTGATGGT |
| HIV_all_2016LANL_WestAfricanGroupM_3571seqs_5873_R_GC_0.38_avg_30.8974_std_6.02042_Tm_28.95 | GTCACATTTTCCA |
| HIV_all_2016LANL_WestAfricanGroupM_3571seqs_5848_R_GC_0.46_avg_30.8974_std_6.02042_Tm_32.10 | CCATTTCCACAAG |
| HIV_all_2016LANL_WestAfricanGroupM_3571seqs_5823_R_GC_0.54_avg_30.8974_std_6.02042_Tm_35.25 | TCAGCAGTCCTTG |
| HIV_all_2016LANL_WestAfricanGroupM_3571seqs_5799_R_GC_0.46_avg_30.8974_std_6.02042_Tm_32.10 | TAGATCTCCTCCT |
| HIV_all_2016LANL_WestAfricanGroupM_3571seqs_5775_R_GC_0.54_avg_30.8974_std_6.02042_Tm_35.25 | ATGTCTCCCACTG |
| HIV_all_2016LANL_WestAfricanGroupM_3571seqs_5751_R_GC_0.23_avg_30.8974_std_6.02042_Tm_22.64 | CTACAATTTAAAG |
| HIV_all_2016LANL_WestAfricanGroupM_3571seqs_5727_R_GC_0.31_avg_30.8974_std_6.02042_Tm_25.79 | TGATAGTGTCATT |
| HIV_all_2016LANL_WestAfricanGroupM_3571seqs_5703_R_GC_0.46_avg_30.8974_std_6.02042_Tm_32.10 | ACTGTCCCTATAG |
| HIV_all_2016LANL_WestAfricanGroupM_3571seqs_5680_R_GC_0.46_avg_30.8974_std_6.02042_Tm_32.10 | CACTAGCTACATG |
| HIV_all_2016LANL_WestAfricanGroupM_3571seqs_5657_R_GC_0.31_avg_30.8974_std_6.02042_Tm_25.79 | ACTTGTTCTTGTA |
| HIV_all_2016LANL_WestAfricanGroupM_3571seqs_5634_R_GC_0.31_avg_30.8974_std_6.02042_Tm_25.79 | ATTAATATCCCCA |
| HIV_all_2016LANL_WestAfricanGroupM_3571seqs_5611_R_GC_0.31_avg_30.8974_std_6.02042_Tm_25.79 | GTCCTATTTCTAT |
| HIV_all_2016LANL_WestAfricanGroupM_3571seqs_5588_R_GC_0.38_avg_30.8974_std_6.02042_Tm_28.95 | CTTCCTTTTCCAT |
| HIV_all_2016LANL_WestAfricanGroupM_3571seqs_5565_R_GC_0.54_avg_30.8974_std_6.02042_Tm_35.25 | TCTGCTCCTGCTT |
| HIV_all_2016LANL_WestAfricanGroupM_3571seqs_5543_R_GC_0.69_avg_30.8974_std_6.02042_Tm_41.56 | GAGCTCCCAGGCT |
| HIV_all_2016LANL_WestAfricanGroupM_3571seqs_5520_R_GC_0.46_avg_30.8974_std_6.02042_Tm_32.10 | CCTAATGGGATGT |
| HIV_all_2016LANL_WestAfricanGroupM_3571seqs_5498_R_GC_0.31_avg_30.8974_std_6.02042_Tm_25.79 | ACTTCTTCTTGTA |
| HIV_all_2016LANL_WestAfricanGroupM_3571seqs_5476_R_GC_0.46_avg_30.8974_std_6.02042_Tm_32.10 | CTAGGGAAATGTC |
| HIV_all_2016LANL_WestAfricanGroupM_3571seqs_5454_R_GC_0.31_avg_30.8974_std_6.02042_Tm_25.79 | CATGGTGTTTAAA |
| HIV_all_2016LANL_WestAfricanGroupM_3571seqs_5432_R_GC_0.31_avg_30.8974_std_6.02042_Tm_25.79 | TATGTCTGGATTT |
| HIV_all_2016LANL_WestAfricanGroupM_3571seqs_5410_R_GC_0.46_avg_30.8974_std_6.02042_Tm_32.10 | CCAACTGCTCTTT |
| HIV_all_2016LANL_WestAfricanGroupM_3571seqs_5388_R_GC_0.54_avg_30.8974_std_6.02042_Tm_35.25 | GCACACACTGCTT |
| HIV_all_2016LANL_WestAfricanGroupM_3571seqs_5367_R_GC_0.15_avg_30.8974_std_6.02042_Tm_19.48 | TTATTTGTTTACA |
| HIV_all_2016LANL_WestAfricanGroupM_3571seqs_5346_R_GC_0.31_avg_30.8974_std_6.02042_Tm_25.79 | CTAACTTCTGTAT |
| HIV_all_2016LANL_WestAfricanGroupM_3571seqs_5325_R_GC_0.69_avg_30.8974_std_6.02042_Tm_41.56 | CCCCGGAAAGTCC |
| HIV_all_2016LANL_WestAfricanGroupM_3571seqs_5283_R_GC_0.15_avg_30.8974_std_6.02042_Tm_19.48 | TAATTGTCAATTT |
| HIV_all_2016LANL_WestAfricanGroupM_3571seqs_5262_R_GC_0.69_avg_30.8974_std_6.02042_Tm_41.56 | GGCCTCCCCTTGT |
| HIV_all_2016LANL_WestAfricanGroupM_3571seqs_5241_R_GC_0.31_avg_30.8974_std_6.02042_Tm_25.79 | GGAGAGTTATATT |
| HIV_all_2016LANL_WestAfricanGroupM_3571seqs_5220_R_GC_0.62_avg_30.8974_std_6.02042_Tm_38.41 | CAGCATCTGAGGG |
| HIV_all_2016LANL_WestAfricanGroupM_3571seqs_5200_R_GC_0.62_avg_30.8974_std_6.02042_Tm_38.41 | AGGCCATCCCACT |
| HIV_all_2016LANL_WestAfricanGroupM_3571seqs_5180_R_GC_0.38_avg_30.8974_std_6.02042_Tm_28.95 | TATCCACCATTGT |
| HIV_all_2016LANL_WestAfricanGroupM_3571seqs_5160_R_GC_0.31_avg_30.8974_std_6.02042_Tm_25.79 | TTATCTATGCCAT |
| HIV_all_2016LANL_WestAfricanGroupM_3571seqs_5140_R_GC_0.38_avg_30.8974_std_6.02042_Tm_28.95 | GTATAGGAATTGG |
| HIV_all_2016LANL_WestAfricanGroupM_3571seqs_5120_R_GC_0.31_avg_30.8974_std_6.02042_Tm_25.79 | TTCTTTCCCTTAT |
| HIV_all_2016LANL_WestAfricanGroupM_3571seqs_5100_R_GC_0.38_avg_30.8974_std_6.02042_Tm_28.95 | ATTTGTCTACAGC |
| HIV_all_2016LANL_WestAfricanGroupM_3571seqs_5080_R_GC_0.31_avg_30.8974_std_6.02042_Tm_25.79 | TTTGTACTACATC |
| HIV_all_2016LANL_WestAfricanGroupM_3571seqs_5060_R_GC_0.23_avg_30.8974_std_6.02042_Tm_22.64 | TTGCTCTTATTTT |
| HIV_all_2016LANL_WestAfricanGroupM_3571seqs_5040_R_GC_0.38_avg_30.8974_std_6.02042_Tm_28.95 | AGGTTAGGATCTA |
| HIV_all_2016LANL_WestAfricanGroupM_3571seqs_5020_R_GC_0.62_avg_30.8974_std_6.02042_Tm_38.41 | CCAGAGAGCTCTC |
| HIV_all_2016LANL_WestAfricanGroupM_3571seqs_5000_R_GC_0.38_avg_30.8974_std_6.02042_Tm_28.95 | GGAACTGAGAAAT |
| HIV_all_2016LANL_WestAfricanGroupM_3571seqs_4981_R_GC_0.46_avg_30.8974_std_6.02042_Tm_32.10 | ACTACTCCTTGAC |
| HIV_all_2016LANL_WestAfricanGroupM_3571seqs_4962_R_GC_0.46_avg_30.8974_std_6.02042_Tm_32.10 | CCACAGTTGAAAC |
| HIV_all_2016LANL_WestAfricanGroupM_3571seqs_4943_R_GC_0.54_avg_30.8974_std_6.02042_Tm_35.25 | AGGGATCCTACCT |
| HIV_all_2016LANL_WestAfricanGroupM_3571seqs_4924_R_GC_0.31_avg_30.8974_std_6.02042_Tm_25.79 | TGTTGCATAGAAT |
| HIV_all_2016LANL_WestAfricanGroupM_3571seqs_4905_R_GC_0.62_avg_30.8974_std_6.02042_Tm_38.41 | CCTGCAAGGCTAG |
| HIV_all_2016LANL_WestAfricanGroupM_3571seqs_4886_R_GC_0.54_avg_30.8974_std_6.02042_Tm_35.25 | CTTTCCTCCTTGG |
| HIV_all_2016LANL_WestAfricanGroupM_3571seqs_4867_R_GC_0.38_avg_30.8974_std_6.02042_Tm_28.95 | TGTAGAGATCCTA |
| HIV_all_2016LANL_WestAfricanGroupM_3571seqs_4848_R_GC_0.38_avg_30.8974_std_6.02042_Tm_28.95 | TTTTGCTCTTCCT |
| HIV_all_2016LANL_WestAfricanGroupM_3571seqs_4811_R_GC_0.46_avg_30.8974_std_6.02042_Tm_32.10 | TGCAATCTGGGTT |
| HIV_all_2016LANL_WestAfricanGroupM_3571seqs_4793_R_GC_0.15_avg_30.8974_std_6.02042_Tm_19.48 | TATATTGATAGAT |
| HIV_all_2016LANL_WestAfricanGroupM_3571seqs_4775_R_GC_0.31_avg_30.8974_std_6.02042_Tm_25.79 | AAAAAGTGCATAC |
| HIV_all_2016LANL_WestAfricanGroupM_3571seqs_4757_R_GC_0.38_avg_30.8974_std_6.02042_Tm_28.95 | CTGTCAAATTTCC |
| HIV_all_2016LANL_WestAfricanGroupM_3571seqs_4739_R_GC_0.46_avg_30.8974_std_6.02042_Tm_32.10 | CAAGGATGTCTTG |
| HIV_all_2016LANL_WestAfricanGroupM_3571seqs_4721_R_GC_0.31_avg_30.8974_std_6.02042_Tm_25.79 | GTACAGTTTAAAG |
| HIV_all_2016LANL_WestAfricanGroupM_3571seqs_4703_R_GC_0.46_avg_30.8974_std_6.02042_Tm_32.10 | TATCTGTCCCTTC |
| HIV_all_2016LANL_WestAfricanGroupM_3571seqs_4686_R_GC_0.46_avg_30.8974_std_6.02042_Tm_32.10 | CCCCAATATGTTC |
| HIV_all_2016LANL_WestAfricanGroupM_3571seqs_4669_R_GC_0.54_avg_30.8974_std_6.02042_Tm_35.25 | CTCTCTCCCATTC |
| HIV_all_2016LANL_WestAfricanGroupM_3571seqs_4652_R_GC_0.46_avg_30.8974_std_6.02042_Tm_32.10 | TTCCCTGTCTTTC |
| HIV_all_2016LANL_WestAfricanGroupM_3571seqs_4635_R_GC_0.69_avg_30.8974_std_6.02042_Tm_41.56 | CACACCTCCCCTG |
| HIV_all_2016LANL_WestAfricanGroupM_3571seqs_4618_R_GC_0.46_avg_30.8974_std_6.02042_Tm_32.10 | TGCCTAACAGCTT |
| HIV_all_2016LANL_WestAfricanGroupM_3571seqs_4601_R_GC_0.46_avg_30.8974_std_6.02042_Tm_32.10 | CCATCTCTTGTCA |
| HIV_all_2016LANL_WestAfricanGroupM_3571seqs_4584_R_GC_0.38_avg_30.8974_std_6.02042_Tm_28.95 | GTATTGCTACTTG |
| HIV_all_2016LANL_WestAfricanGroupM_3571seqs_4568_R_GC_0.54_avg_30.8974_std_6.02042_Tm_35.25 | TCTCCTACTGGGA |
| HIV_all_2016LANL_WestAfricanGroupM_3571seqs_4552_R_GC_0.38_avg_30.8974_std_6.02042_Tm_28.95 | GGCACTACTTTTA |
| HIV_all_2016LANL_WestAfricanGroupM_3571seqs_4536_R_GC_0.38_avg_30.8974_std_6.02042_Tm_28.95 | TGATAGTGCCATT |
| HIV_all_2016LANL_WestAfricanGroupM_3571seqs_4520_R_GC_0.38_avg_30.8974_std_6.02042_Tm_28.95 | CAAGGATATCTTG |
| HIV_all_2016LANL_WestAfricanGroupM_3571seqs_4504_R_GC_0.69_avg_30.8974_std_6.02042_Tm_41.56 | CTCTCCCTGGTCC |
| HIV_all_2016LANL_WestAfricanGroupM_3571seqs_4488_R_GC_0.38_avg_30.8974_std_6.02042_Tm_28.95 | TAAAACTCCGGAT |
| HIV_all_2016LANL_WestAfricanGroupM_3571seqs_4473_R_GC_0.23_avg_30.8974_std_6.02042_Tm_22.64 | ACAAATTTCTGTT |
| HIV_all_2016LANL_WestAfricanGroupM_3571seqs_4458_R_GC_0.54_avg_30.8974_std_6.02042_Tm_35.25 | CTGTCAAACCTCC |
| HIV_all_2016LANL_WestAfricanGroupM_3571seqs_4443_R_GC_0.38_avg_30.8974_std_6.02042_Tm_28.95 | CACAATTGAAGCA |
| HIV_all_2016LANL_WestAfricanGroupM_3571seqs_4428_R_GC_0.69_avg_30.8974_std_6.02042_Tm_41.56 | GCGGAAAGTCCCC |
| HIV_all_2016LANL_WestAfricanGroupM_3571seqs_4413_R_GC_0.46_avg_30.8974_std_6.02042_Tm_32.10 | CTTTGACCACTTG |
| HIV_all_2016LANL_WestAfricanGroupM_3571seqs_4398_R_GC_0.38_avg_30.8974_std_6.02042_Tm_28.95 | TATAGAAAAGCGC |
| HIV_all_2016LANL_WestAfricanGroupM_3571seqs_4383_R_GC_0.23_avg_30.8974_std_6.02042_Tm_22.64 | TTCTGTATATCAT |
| HIV_all_2016LANL_WestAfricanGroupM_3571seqs_4368_R_GC_0.54_avg_30.8974_std_6.02042_Tm_35.25 | GTGGAAAGTCCCT |
| HIV_all_2016LANL_WestAfricanGroupM_3571seqs_4353_R_GC_0.62_avg_30.8974_std_6.02042_Tm_38.41 | AGCCAGGCACAAG |
| HIV_all_2016LANL_WestAfricanGroupM_3571seqs_4338_R_GC_0.46_avg_30.8974_std_6.02042_Tm_32.10 | CCTGCTGTGTTTT |
| HIV_all_2016LANL_WestAfricanGroupM_3571seqs_4323_R_GC_0.38_avg_30.8974_std_6.02042_Tm_28.95 | CTACTGCTCTTTT |
| HIV_all_2016LANL_WestAfricanGroupM_3571seqs_4308_R_GC_0.46_avg_30.8974_std_6.02042_Tm_32.10 | TGTCAAACTGCCA |
| HIV_all_2016LANL_WestAfricanGroupM_3571seqs_4293_R_GC_0.46_avg_30.8974_std_6.02042_Tm_32.10 | AAATTTCCTGGCC |
| HIV_all_2016LANL_WestAfricanGroupM_3571seqs_4278_R_GC_0.62_avg_30.8974_std_6.02042_Tm_38.41 | TCCCCAAGTGCTG |
| HIV_all_2016LANL_WestAfricanGroupM_3571seqs_4264_R_GC_0.38_avg_30.8974_std_6.02042_Tm_28.95 | TCTCTATCCCATT |
| HIV_all_2016LANL_WestAfricanGroupM_3571seqs_4250_R_GC_0.23_avg_30.8974_std_6.02042_Tm_22.64 | CTTTAATACTTCT |
| HIV_all_2016LANL_WestAfricanGroupM_3571seqs_4236_R_GC_0.15_avg_30.8974_std_6.02042_Tm_19.48 | TGTTATTACTAAT |
| HIV_all_2016LANL_WestAfricanGroupM_3571seqs_4222_R_GC_0.54_avg_30.8974_std_6.02042_Tm_35.25 | CCTGCCAATAGTC |
| HIV_all_2016LANL_WestAfricanGroupM_3571seqs_4208_R_GC_0.31_avg_30.8974_std_6.02042_Tm_25.79 | AAATAAGCATCTC |
| HIV_all_2016LANL_WestAfricanGroupM_3571seqs_4194_R_GC_0.31_avg_30.8974_std_6.02042_Tm_25.79 | CTATATATCCACT |
| HIV_all_2016LANL_WestAfricanGroupM_3571seqs_4180_R_GC_0.54_avg_30.8974_std_6.02042_Tm_35.25 | CCCTATCTGTCCA |
| HIV_all_2016LANL_WestAfricanGroupM_3571seqs_4166_R_GC_0.31_avg_30.8974_std_6.02042_Tm_25.79 | GTCAAATTAGCAT |
| HIV_all_2016LANL_WestAfricanGroupM_3571seqs_4153_R_GC_0.15_avg_30.8974_std_6.02042_Tm_19.48 | TCTATATATACTA |
| HIV_all_2016LANL_WestAfricanGroupM_3571seqs_4139_R_GC_0.62_avg_30.8974_std_6.02042_Tm_38.41 | TCCCCTCCTGAGT |
| HIV_all_2016LANL_WestAfricanGroupM_3571seqs_4125_R_GC_0.54_avg_30.8974_std_6.02042_Tm_35.25 | GGGATTGTGATGG |
| HIV_all_2016LANL_WestAfricanGroupM_3571seqs_4112_R_GC_0.31_avg_30.8974_std_6.02042_Tm_25.79 | ATCAGTGCAATTT |
| HIV_all_2016LANL_WestAfricanGroupM_3571seqs_4086_R_GC_0.46_avg_30.8974_std_6.02042_Tm_32.10 | TCCCCAAGATCAT |
| HIV_all_2016LANL_WestAfricanGroupM_3571seqs_4073_R_GC_0.62_avg_30.8974_std_6.02042_Tm_38.41 | AGTTCCCTGGCCT |
| HIV_all_2016LANL_WestAfricanGroupM_3571seqs_4060_R_GC_0.31_avg_30.8974_std_6.02042_Tm_25.79 | TCAAGCCTATAAA |
| HIV_all_2016LANL_WestAfricanGroupM_3571seqs_4047_R_GC_0.46_avg_30.8974_std_6.02042_Tm_32.10 | ACTGTACCTACAG |
| HIV_all_2016LANL_WestAfricanGroupM_3571seqs_4034_R_GC_0.38_avg_30.8974_std_6.02042_Tm_28.95 | CTTCTGTTGTTTC |
| HIV_all_2016LANL_WestAfricanGroupM_3571seqs_4021_R_GC_0.54_avg_30.8974_std_6.02042_Tm_35.25 | TTCTGAGAGGGAG |
| HIV_all_2016LANL_WestAfricanGroupM_3571seqs_4008_R_GC_0.31_avg_30.8974_std_6.02042_Tm_25.79 | GTTACATTTTCCA |
| HIV_all_2016LANL_WestAfricanGroupM_3571seqs_3995_R_GC_0.54_avg_30.8974_std_6.02042_Tm_35.25 | CCCTGCTTCTGTA |
| HIV_all_2016LANL_WestAfricanGroupM_3571seqs_3982_R_GC_0.38_avg_30.8974_std_6.02042_Tm_28.95 | CTTCTGCTAAACT |
| HIV_all_2016LANL_WestAfricanGroupM_3571seqs_3969_R_GC_0.46_avg_30.8974_std_6.02042_Tm_32.10 | GGGATTGTGATGT |
| HIV_all_2016LANL_WestAfricanGroupM_3571seqs_3956_R_GC_0.46_avg_30.8974_std_6.02042_Tm_32.10 | ATCCCTGCCTAAT |
| HIV_all_2016LANL_WestAfricanGroupM_3571seqs_3943_R_GC_0.46_avg_30.8974_std_6.02042_Tm_32.10 | TCTACTGGATCCA |
| HIV_all_2016LANL_WestAfricanGroupM_3571seqs_3930_R_GC_0.31_avg_30.8974_std_6.02042_Tm_25.79 | CATATATTGGTAG |
| HIV_all_2016LANL_WestAfricanGroupM_3571seqs_3918_R_GC_0.31_avg_30.8974_std_6.02042_Tm_25.79 | ATTAATTCAGCCT |
| HIV_all_2016LANL_WestAfricanGroupM_3571seqs_3906_R_GC_0.54_avg_30.8974_std_6.02042_Tm_35.25 | CCATGTCCACAAG |
| HIV_all_2016LANL_WestAfricanGroupM_3571seqs_3894_R_GC_0.54_avg_30.8974_std_6.02042_Tm_35.25 | CCCTACCTGACTA |
| HIV_all_2016LANL_WestAfricanGroupM_3571seqs_3882_R_GC_0.38_avg_30.8974_std_6.02042_Tm_28.95 | TCAGTGCAGTTTA |
| HIV_all_2016LANL_WestAfricanGroupM_3571seqs_3870_R_GC_0.31_avg_30.8974_std_6.02042_Tm_25.79 | GTATTGGTAGATA |
| HIV_all_2016LANL_WestAfricanGroupM_3571seqs_3858_R_GC_0.23_avg_30.8974_std_6.02042_Tm_22.64 | TTCTATGAATACT |
| HIV_all_2016LANL_WestAfricanGroupM_3571seqs_3846_R_GC_0.31_avg_30.8974_std_6.02042_Tm_25.79 | TTCTGTTGTTTGT |
| HIV_all_2016LANL_WestAfricanGroupM_3571seqs_3834_R_GC_0.54_avg_30.8974_std_6.02042_Tm_35.25 | CCACTTGCCACAT |
| HIV_all_2016LANL_WestAfricanGroupM_3571seqs_3822_R_GC_0.23_avg_30.8974_std_6.02042_Tm_22.64 | TTATAGTGTCATT |
| HIV_all_2016LANL_WestAfricanGroupM_3571seqs_3810_R_GC_0.69_avg_30.8974_std_6.02042_Tm_41.56 | TTGGCCACTCCCC |
| HIV_all_2016LANL_WestAfricanGroupM_3571seqs_3798_R_GC_0.46_avg_30.8974_std_6.02042_Tm_32.10 | TCCTGGTCCTATA |
| HIV_all_2016LANL_WestAfricanGroupM_3571seqs_3786_R_GC_0.31_avg_30.8974_std_6.02042_Tm_25.79 | CTACAGTTTAAAG |
| HIV_all_2016LANL_WestAfricanGroupM_3571seqs_3774_R_GC_0.38_avg_30.8974_std_6.02042_Tm_28.95 | TGTCAGCAATCTT |
| HIV_all_2016LANL_WestAfricanGroupM_3571seqs_3762_R_GC_0.23_avg_30.8974_std_6.02042_Tm_22.64 | ATTTTTCCTTCTT |
| HIV_all_2016LANL_WestAfricanGroupM_3571seqs_3750_R_GC_0.31_avg_30.8974_std_6.02042_Tm_25.79 | TTCTTTCTCTTAC |
| HIV_all_2016LANL_WestAfricanGroupM_3571seqs_3738_R_GC_0.62_avg_30.8974_std_6.02042_Tm_38.41 | CCACTGGCCACAT |
| HIV_all_2016LANL_WestAfricanGroupM_3571seqs_3727_R_GC_0.38_avg_30.8974_std_6.02042_Tm_28.95 | CTATCAAACTTCC |
| HIV_all_2016LANL_WestAfricanGroupM_3571seqs_3716_R_GC_0.62_avg_30.8974_std_6.02042_Tm_38.41 | ACACAGAGTGGGG |
| HIV_all_2016LANL_WestAfricanGroupM_3571seqs_3694_R_GC_0.31_avg_30.8974_std_6.02042_Tm_25.79 | TCTATTCCATCTA |
| HIV_all_2016LANL_WestAfricanGroupM_3571seqs_3683_R_GC_0.46_avg_30.8974_std_6.02042_Tm_32.10 | TCCCTGATGCTAA |
| HIV_all_2016LANL_WestAfricanGroupM_3571seqs_3672_R_GC_0.31_avg_30.8974_std_6.02042_Tm_25.79 | TATTCATGGATTC |
| HIV_all_2016LANL_WestAfricanGroupM_3571seqs_3661_R_GC_0.15_avg_30.8974_std_6.02042_Tm_19.48 | TTTGTATATCATT |
| HIV_all_2016LANL_WestAfricanGroupM_3571seqs_3650_R_GC_0.46_avg_30.8974_std_6.02042_Tm_32.10 | ATCCTTGTGTGTG |
| HIV_all_2016LANL_WestAfricanGroupM_3571seqs_3639_R_GC_0.15_avg_30.8974_std_6.02042_Tm_19.48 | TATTGTCTTATTA |
| HIV_all_2016LANL_WestAfricanGroupM_3571seqs_3628_R_GC_0.54_avg_30.8974_std_6.02042_Tm_35.25 | AGCCAGGACTCTT |
| HIV_all_2016LANL_WestAfricanGroupM_3571seqs_3618_R_GC_0.62_avg_30.8974_std_6.02042_Tm_38.41 | CCTCCATCACGTG |
