# Supplementary material for: New Genomes from the Congo Basin Expand History of CRF01_AE Origin and Dissemination
Source: AIDS Res Hum Retroviruses. 2020 Jul 2;36(7):574–82. doi: 10.1089/aid.2020.0031 (PMC7398440; doi:10.1089/aid.2020.0031)
Supplement: Supplemental data [file Supp_Fig1.pdf]

Supplementary Data

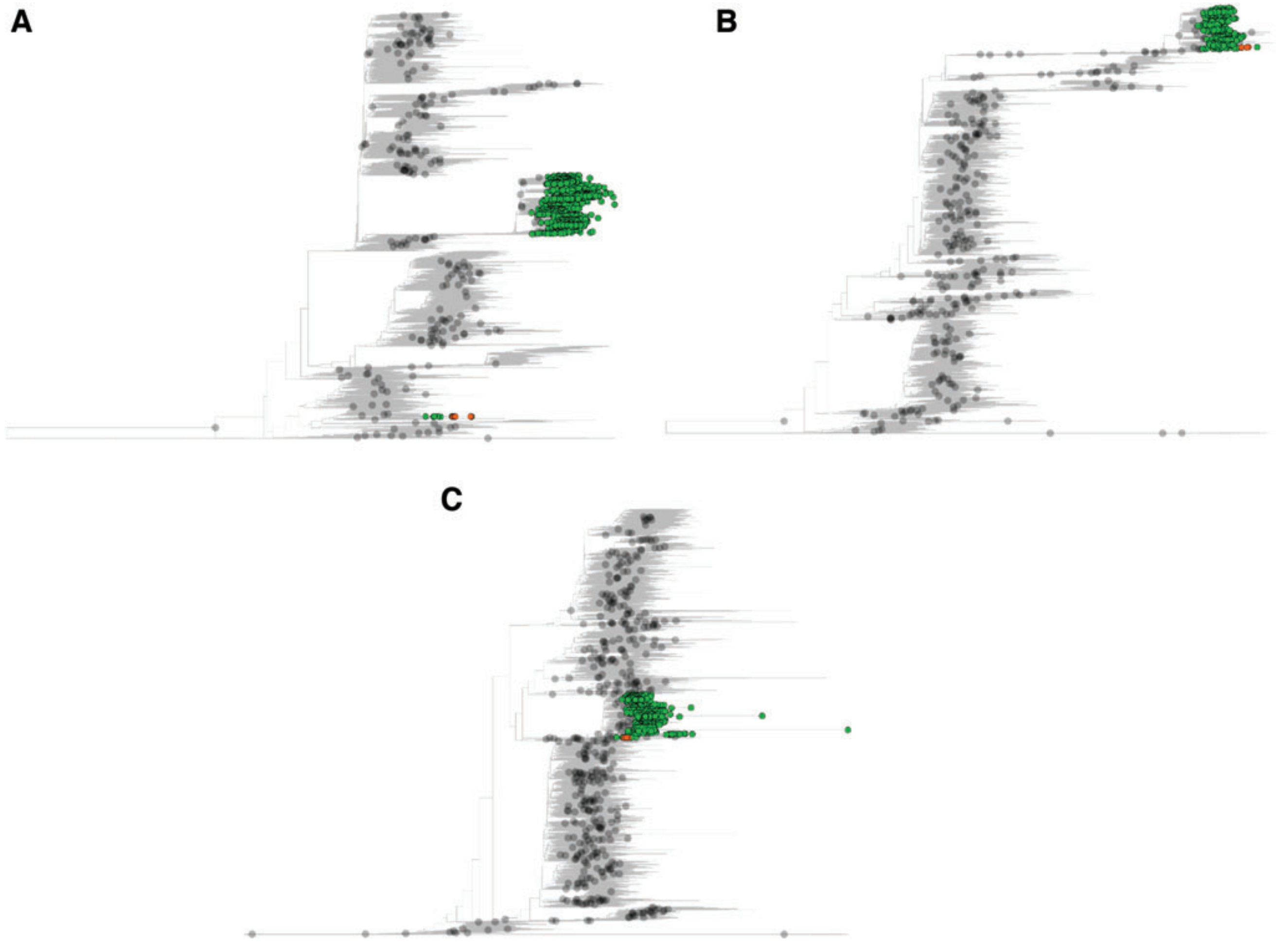

SUPPLEMENTARY FIG. S1. Maximum-likelihood phylogenetic analysis of HIV-1 *gag*, *pol*, and *env* subgenomic regions, including reference sequences collected from the LANL HIV database in addition to the four CRF01_AE sequences described in this study (U7957, U8216, 234-40, and 1002-28). Trees were reconstructed with the general time-reversible nucleotide substitution model with gamma distribution among site rate heterogeneity. SH test on the three alternate topologies (NNIs) was used to estimate the reliability of each split in the tree and the *gray circles* identify clades with SH = 1. *Orange dots* on terminal nodes identify those sequences described in this study and *green dots* indicate CRF01_AE reference samples. **(A)** Phylogenetic tree, including 9,431 LANL HIV database *gag* reference sequences *(nucleotide positions relative to HXB2 genome start: 790–2,288)*. CRF01_AE sequences were split into two clades: (i) the major clade includes 1,279 sequences and (ii) the minor clade is composed by 12 samples and includes all three ancestral reference sequences (sampled in 1990). **(B)** Maximum-likelihood phylogenetic analysis, including 5,119 HIV-1 *pol* reference sequences (nucleotide positions relative to HXB2 genome start: 2,085–5,093). **(C)** Phylogenetic tree, including 6,966 LANL HIV database *env* reference sequences (nucleotide positions relative to HXB2 genome start: 6,225–8,795). CRF, circulating recombinant form; LANL, Los Alamos National Laboratory; NNIs, nearest neighbor interchanges; SH, Shimodaira-Hasegawa.
