# Supplementary material for: New Genomes from the Congo Basin Expand History of CRF01_AE Origin and Dissemination
Source: AIDS Res Hum Retroviruses. 2020 Jul 2;36(7):574–82. doi: 10.1089/aid.2020.0031 (PMC7398440; doi:10.1089/aid.2020.0031)
Supplement: Supplemental data [file Supp_Fig2.pdf]

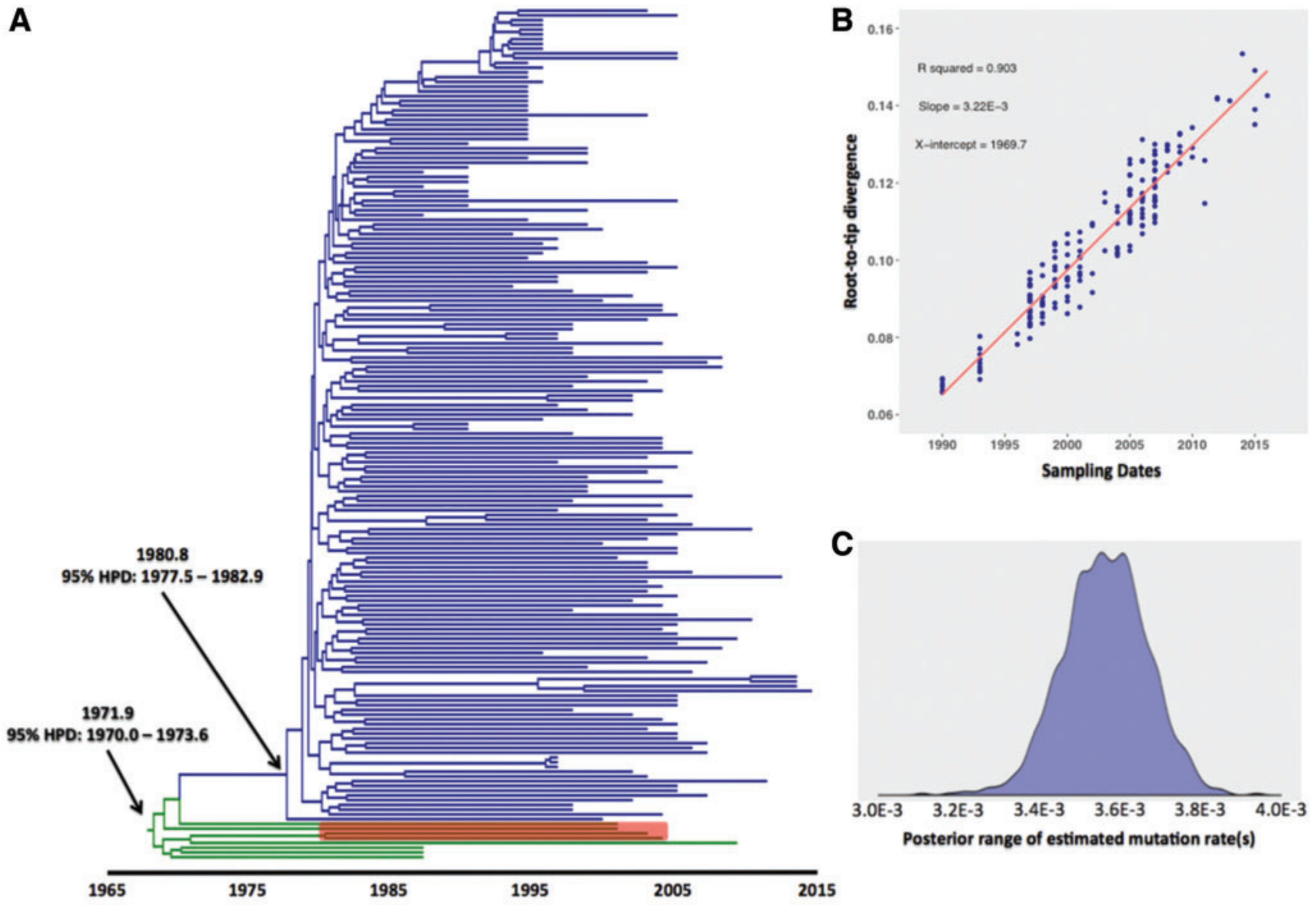

SUPPLEMENTARY FIG. S2. Bayesian analysis comprising 179 CRF01_AE sequences under a skyride prior for modeling the population size changes through time. **(A)** Bayesian phylogenetic tree showing the estimated years for the arising of CRF01_AE and the expansion from Africa. Branches highlighted in *red* denote the CRF01_AE described in this study, *green branches* indicate sequences sampled in Africa, and *blue branches* show sequences isolated outside Africa. **(B)** Molecular signal for the 179 CRF01_AE sequences. **(C)** Posterior range for the estimated evolutionary rate after 50% burn-in was removed. HPD, highest posterior density.
